# Stage-specific requirement for METTL3-dependent m^6^A epitranscriptomic regulation during myogenesis

**DOI:** 10.1038/s42003-025-08759-5

**Published:** 2025-08-30

**Authors:** Ye-Ya Tan, Yang-Wen Ou, Qin Zuo, Yi Luo, Wei-Cai Chen, Wan-Xin Chen, Xin-Wang Zhi, Pei-Wen Lin, Jia-Xing Lu, Peng Liu, Si-Min Liang, Qing-Hai Lian, Lian-Dong Zuo, Hong-Wen Xu, Shu-Juan Xie

**Affiliations:** 1 GMU-GIBH Joint School of Life Science, The Guangdong-Hong Kong-Macao Joint Laboratory for Cell Fate Regulation and Diseases, Guangzhou Medical University, Guangzhou, China; 2 Department of Cardiovascular Medicine, Second Affiliated Hospital of Guangzhou, University of Chinese Medicine, Guangzhou, China; 3 Jiaxing Kaiyi Hospital, Economic and Technological Development Zone, Jiaxing, Zhejiang Province China; 4 Vaccine Research Institute of Sun Yat-sen University, The Third Affiliated Hospital of Sun Yat-sen University, Guangzhou, China; 5 Biotherapy Center, The Third Affiliated Hospital of Sun Yat-sen University, Guangzhou, China; 6 Guangdong Institute of Intelligence Science and Technology, Hengqin, Zhuhai, Guangdong, China

**Keywords:** Computational biology and bioinformatics, RNA metabolism

## Abstract

The regulatory role of N^6^-methyladenosine (m^6^A) modification in skeletal muscle myogenesis and muscle homeostasis remains poorly characterized, particularly regarding the functional significance of methyltransferase-like 3 (METTL3), the catalytic subunit of the m^6^A methyltransferase complex (MTC), in myogenic regulation. Through systematic investigation of m^6^A epitranscriptomic remodeling during myogenesis, we demonstrate that METTL3-mediated m^6^As orchestrates myoblast fusion processes in both differentiation and regeneration contexts. Notably, we observed marked induction of *Mettl3* expression post-injury, accompanied by substantial transcriptomic alterations in myogenesis-related pathways. High-resolution m^6^A mapping revealed distinct dynamic patterns of METTL3-regulated m^6^As during differentiation, exhibiting dichotomous regulation across target transcripts. Mechanistically, we identified myogenic fusion factors *Mymx* and *Mymk* as direct targets of METTL3, showing concomitant upregulation of both transcript abundance and m^6^A deposition during myogenesis. This study provides comprehensive multi-omics resources delineating the mechanistic landscape of METTL3-regulated m^6^As in myogenic programming, establishing METTL3 as a critical regulatory node governing myoblast fusion dynamic.

## Abstract (web summary)

Function and mechanism underlying m^6^A modifications in myoblast differentiation and skeletal muscle regeneration while elucidating the regulatory role of METTL3 in promoting successful myoblast fusion.

## Introduction

N6-methyladenosine (m^6^A) is the most prevalent internal RNA modification in mRNAs and is highly conserved in mammals and other eukaryotic species^1,2^. It is primarily introduced by the methyltransferase complex (MTC), the core component of which is a heterodimer composed of methyltransferase-like 3 (METTL3) and methyltransferase-like 14 (METTL14)^3–5^. Among these subunits, METTL3 exhibits catalytic activity. Sequence analysis revealed that m^6^A modification typically occurs within the consensus motif RRACH (R = G or A; H = A, C, or U; where A is converted to m^6^A). The distribution of m^6^A methylation on the RRACH motif is nonrandom throughout the transcript, particularly within the coding sequence (CDS), 3ʹ untranslated region (3ʹ UTR), and around the stop codon. This dynamic and reversible m^6^A modification governs mRNA stability, splicing, nuclear export, and translation and plays crucial roles in various biological processes, such as embryonic development and regeneration^6^.

The involvement of m^6^A in muscle formation, maintenance of muscle homeostasis, and its multifaceted functions in musculoskeletal disorders has been demonstrated. Following skeletal muscle injury, an increase in global m^6^A levels is observed that corresponds to the rapid proliferation of muscle stem cells (MuSCs). Additionally, primary mouse myoblasts or C2C12 myoblasts exhibit elevated global m^6^A levels during proliferation and a subsequent decline in m^6^A levels during differentiation in vitro^7^. Although *Mettl3* knockdown impacts the engraftment of MuSCs following primary transplantation, further investigations are warranted to comprehensively elucidate the key downstream targets and underlying mechanisms involved. For example, METTL3 facilitates the expression of the MEF2C protein through posttranscriptional modification in an m^6^A-YTHDF1-dependent manner^8^. METTL3-mediated stabilization of processed *Myod* through m^6^A modification of the 5’ UTR contributes to maintaining myogenic potential during proliferative phases^9^. METTL3 also regulates skeletal muscle size during hypertrophy by inhibiting the synthesis of activin type 2 A receptor (ACVR2A) and attenuating the activation of antihypertrophic signals^10^. Additionally, recent evidence suggests heightened sensitivity to m^6^A deletion and *Mettl3* downregulation in skeletal muscles during aging^11^. A comprehensive understanding of the function and mechanism of METTL3 in skeletal muscle generation is imperative for a profound understanding and effective development of treatments targeting skeletal muscle diseases, including skeletal muscle injury and aging.

Skeletal muscle regeneration plays a critical role in delaying the loss of functional skeletal muscle; however, dysregulation of genes associated with myogenesis can hinder this process and subsequently impact its functionality, leading to various detrimental consequences^12,13^. Efficient myogenesis relies on the timely regulation of gene expression. Posttranscriptional regulation can coordinate rapid changes in RNA or protein levels without altering transcription^14,15^. In this study, we investigated distinct patterns of m^6^A modifications in various genes during skeletal muscle differentiation, with some exhibiting increased levels and others showing decreased levels. Notably, our findings identify METTL3 as a pivotal regulator responsible for orchestrating these modifications. Moreover, we identified specific subsets of long noncoding RNAs associated with these genes. Additionally, during CTX-induced skeletal muscle injury, we observed significant *Mettl3* upregulation and detected numerous alterations in myogenic pathways and genes linked to its expression. Among them, *Mymx* and *Mymk* emerge as crucial components downstream of METTL3 involved in myogenesis, their expression levels and m^6^A modifications increase during this process. Overall, our study provides valuable insights into the function and mechanism underlying m^6^A modifications in myoblast differentiation and skeletal muscle regeneration while elucidating the regulatory role of METTL3 in promoting successful myoblast fusion, which is a critical step toward effective myogenesis.

## Results

### Identification and integrative analysis of DEGs during skeletal muscle regeneration

**Fig. 1:**
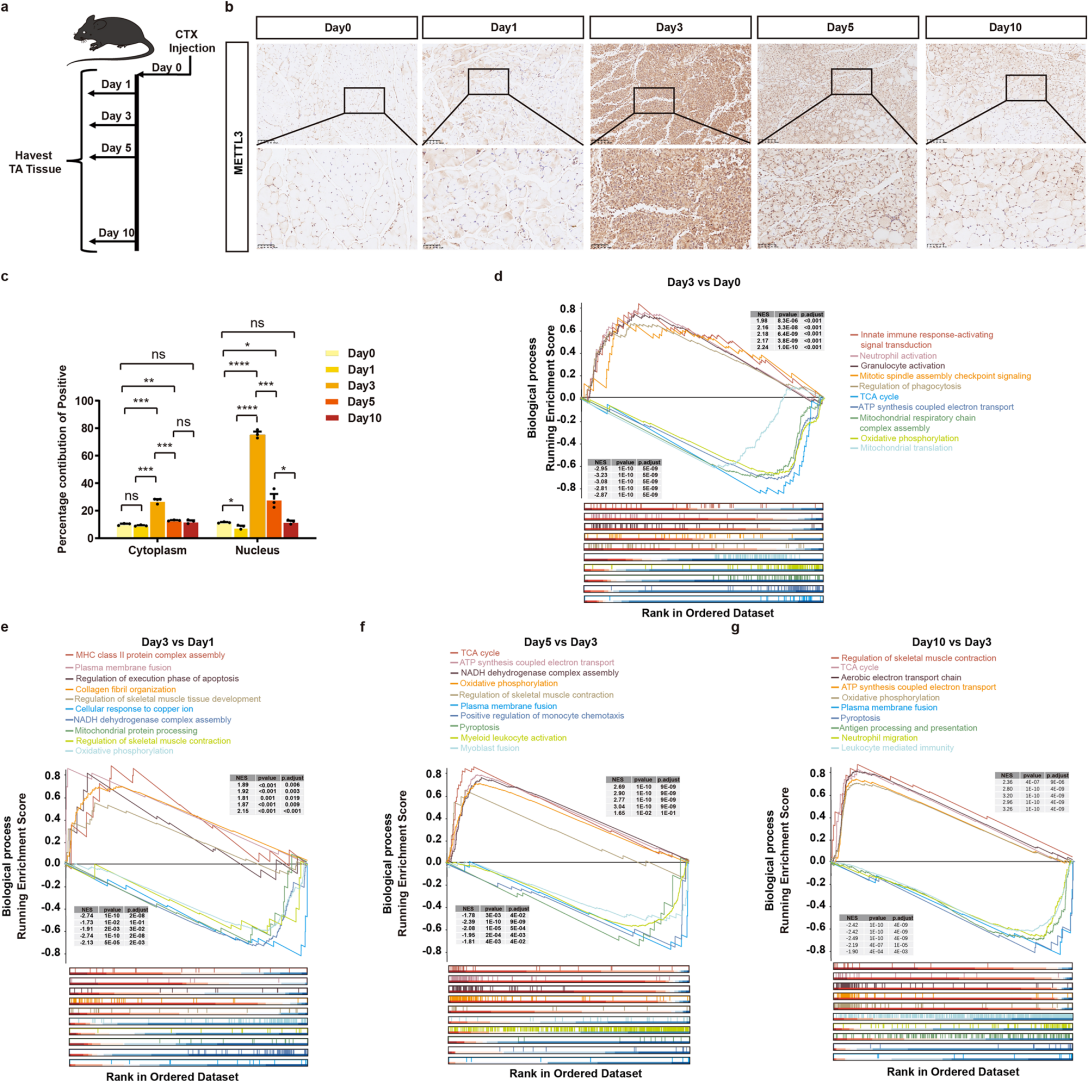
Identification and integrative analysis of DEGs during skeletal muscle regeneration. **a** Timeline characterizing the skeletal muscle repair model. **b** Representative immunohistochemistry of METTL3 from TA muscles at 1-, 3-, 5-, and 10-days following CTX-induced skeletal muscle injury (*n* = 3). Scale bars, 100 μm. **c** Quantification of anti-METTL3 staining intensity in immunohistochemistry from panel (**b**). **d** GSEA analysis on the entire set of DEGs between the third day post-injury and pre-injury conditions. **e** GSEA analysis on the entire set of DEGs between the first- and third-day post-injury. **f** GSEA analysis on the entire set of DEGs between day 3 and day 5 post-injury. **g** GSEA analysis on the entire set of DEGs between day 3 and day 10 post-injury. Data presented as means ± SEM. ns. not significant, *P < 0.05, **P < 0.01, and ***P < 0.001, by two-sided Student’s *t* test.

To identify METTL3 involved in skeletal muscle regeneration, we utilized adult mice to construct a skeletal muscle repair model. We collected the tibialis anterior (TA) muscles of healthy adult mice at 1, 3, 5, and 10 days after injection of CTX to induce myofiber damage (Fig. 1a). The resolution of inflammation gradually occurs concomitantly with the progression of skeletal muscle repair, leading to a gradual recovery in skeletal muscle morphology^16^. The regulation of skeletal muscle injury repair is governed by a multitude of myogenic regulatory factors. Hence, we investigated METTL3 expression levels throughout the process and observed significant upregulation of METTL3 expression on day 3 following skeletal muscle injury (Fig. 1b, c). To further elucidate the differentially expressed genes (DEGs) involved in skeletal muscle regeneration following injury, we conducted RNA-Seq analysis at multiple time points throughout the regenerative process (Supplementary Fig. 1 and Supplementary Data 1). Additionally, GSEA was performed on the entire set of genes ( | NES | > 1, adjusted p value < 0.05, q value < 0.25) (Supplementary Data 2). Three days after skeletal muscle injury, the genes involved in this process were enriched in 1133 pathways associated with biological processes, with the most notably upregulated pathways including innate immune response-activating signal transduction, neutrophil activation, granulocyte activation and mitotic spindle assembly checkpoint signaling. Conversely, the pathways exhibiting the most significant downregulation included the citrate cycle (TCA cycle), ATP synthesis coupled electron transport and oxidative phosphorylation (Fig. 1d). During the period from day 1 to day 3 after skeletal muscle injury, an enrichment of genes involved in this process was observed across a total of 462 pathways associated with diverse biological processes (Fig. 1e). The significantly upregulated pathways included MHC class II protein complex assembly, plasma membrane fusion, regulation of the execution phase of apoptosis, and collagen fibril organization. Notably, the downregulated pathways included the cellular response to copper ions, NADH dehydrogenase complex assembly, mitochondrial protein processing, and the regulation of skeletal muscle contraction. The subsequent step involved conducting GSEA on genes whose expression changed from day 3 to either day 5 or day 10 following skeletal muscle injury, and the majority of the enriched pathways for which expression was upregulated or downregulated within three days after injury were reversed at both time points (Fig. 1f, g). These findings align with the observed changes in cellular dynamics following skeletal muscle injury^17^. Normal skeletal muscle is endowed with sufficient nutrients and oxygen through an intricate network of blood vessels^18^. Under resting conditions in adults, minimal or no mitotic activity is observed in muscle tissue. However, following injury, these cells proliferate and disperse throughout muscle tissue along with infiltrating immune cells^19^. In the early stage of the inflammatory response following skeletal muscle injury, macrophages play crucial roles in clearing tissue debris and releasing cytokines, as well as growth factors that stimulate satellite cell proliferation and differentiation^20^. During the process of muscle regeneration, satellite cells become activated, and some eventually upregulate transcription factors that initiate the myogenic differentiation program^21^. Once differentiated into mature muscle cells, these cells align themselves and either form new syncytial muscle fibers or fuse with existing fibers. Upon completion of this regenerative response, homeostasis is restored within the tissue, and resident cell populations return to their resting state. The upregulated or downregulated KEGG pathways after skeletal muscle injury were also subjected to analysis (Supplementary Fig. 2).

**Fig. 2:**
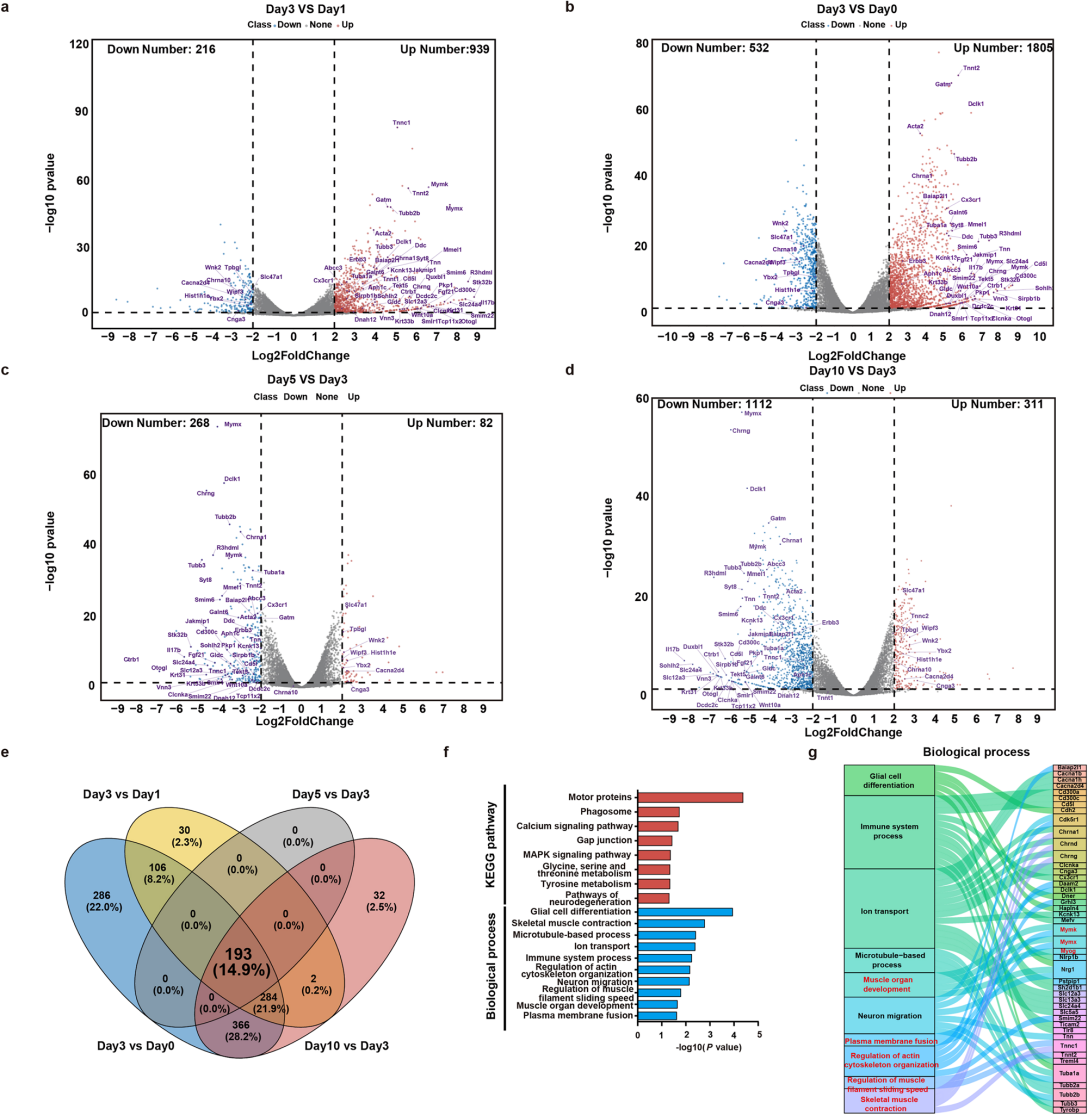
Comparative analysis of gene expression levels on the third day post-injury relative to those at other time points. **a** Volcano plot of DEGs between the first- and third-day post-injury. **b** Volcano plot of DEGs between the third day post-injury and pre-injury conditions. **c** Volcano plot of DEGs between day 3 and day 5 post-injury. **d** Volcano plot of DEGs between day 3 and day 10 post-injury. **e** Venn diagram showing the proportion of DEGs between the four comparison groups. **f** KEGG and GO analysis of intersection of the overlapping genes. **g** The Sankey map illustrates the GO pathway of TOP genes enrichment.

We subsequently performed a comprehensive comparative analysis and identified substantial alterations in gene expression levels on the third day post-injury relative to pre-injury levels (Fig. 2a–d). To elucidate the key effector factors associated with METTL3 in skeletal muscle regeneration, we performed a Venn diagram analysis across four comparison groups, thereby successfully identifying 193 overlapping genes (Fig. 2e). The expression patterns of these DEGs exhibited a reversal before and after the third day of skeletal muscle regeneration, serving as the designated time point. To further visualize the expression patterns of the top DEGs, we annotated a selection of top genes and plotted them on a volcano map. To gain a comprehensive understanding of the biological processes and pathways associated with these overlapping DEGs, Gene Ontology (GO) and Kyoto Encyclopedia of Genes and Genomes (KEGG) analyses were performed (Fig. 1f, Supplementary Data 3). KEGG analysis revealed that these genes were predominantly enriched in the calcium signaling pathway, the MAPK signaling pathway and other pathways associated with myogenesis (Supplementary Fig. 3)^22–24^. In the context of GO analysis, a total of 52 GO terms associated with biological processes were identified. We subsequently conducted further analysis of the GO pathways enriched with the top genes and generated Sankey diagrams. These top genes were enriched predominantly in pathways related to the inflammatory response and myogenesis. Notably, plasma membrane fusion and the regulation of actin cytoskeleton organization, which are crucial pathways for myoblast fusion, were featured prominently in the list (Fig. 2g). The expression levels of the top genes in this group of enriched pathways at different time points were visualized with a chord diagram (Supplementary Fig. 3).

In summary, substantial dynamic changes in the expression levels of numerous genes are noted during skeletal muscle regeneration following injury. Notably, during the initial phase of skeletal muscle injury, along with the progression of inflammation, gradual upregulation of the cell fusion pathway involved in early myogenesis is observed. Furthermore, *Mettl3* expression is significantly upregulated throughout this process.

### Analysis of the role of METTL3 in myoblast differentiation and fusion processes

**Fig. 3:**
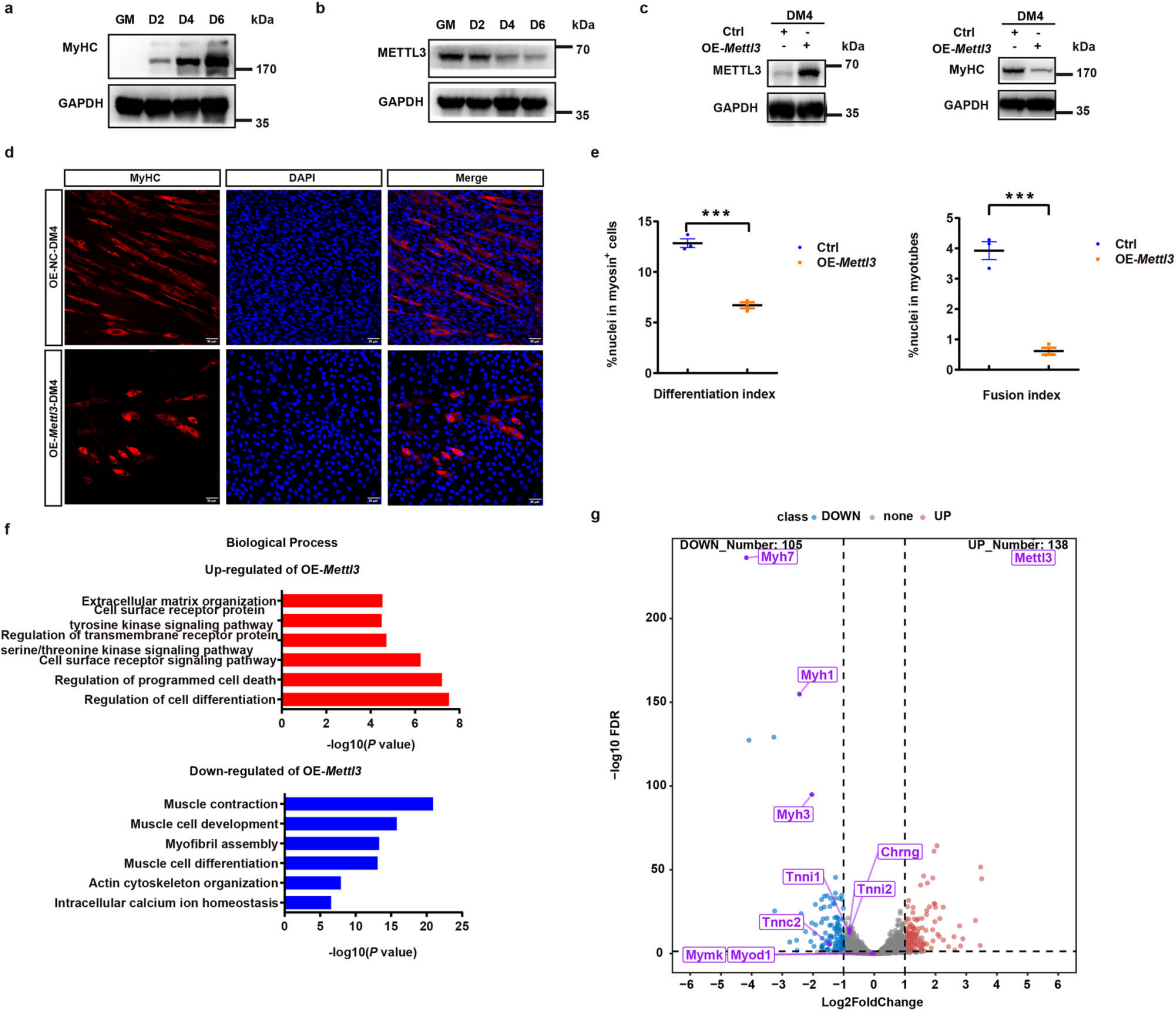
The impact of *Mettl3* overexpression on myoblast differentiation and fusion. **a** Immunoblotting analysis of MyHC during myoblasts differentiation. **b** Immunoblotting analysis of METTL3 during myoblasts differentiation. **c** Immunoblotting analysis of METTL3 and MyHC in *Mettl3*-overexpressing cells and GFP-overexpressing cells. GFP-overexpressing cells were used as negative controls. **d** Representative immunofluorescent staining of *Mettl3* overexpressed cells and wildtype cells on the fourth day post-differentiation (*n* = 3). Red indicated MyHC; blue indicated DAPI staining of nuclei. The merged images were shown. Scale bars, 50 μm. **e** Differentiation index was quantified from representative immunofluorescent images. The differentiation index is defined as the percentage of MyHC-positive cells relative to the total number of nuclei. The fusion index is defined as the ratio of the number of myotubes, characterized by MyHC-positive cells containing at least two nuclei, to the total number of nuclei in the field. **f** GO analysis of DEGs (up-regulated or down-regulated) in *Mettl3*-overexpressing cells. **g** Volcano plot of DEGs between *Mettl3*-overexpressing cells and GFP-overexpressing cells on the fourth day post-differentiation. Data presented as means ± SEM. ns., not significant, *P < 0.05, **P < 0.01, and ***P < 0.001, by two-sided Student’s *t* test, GAPDH was used as the internal control.

We further investigated the role of METTL3 in myoblast differentiation. To assess skeletal muscle differentiation, we utilized mouse C2C12 myoblasts as an in vitro model for studying myogenesis. The expansion of C2C12 myoblasts was performed in the presence of serum, followed by the induction of differentiation through serum withdrawal. METTL3 levels decreased during C2C12 cell differentiation (Fig. 3a, b). Using C2C12 cells, we established stable cell lines overexpressing *Mettl3* and employed immunoblotting analysis and immunofluorescence analysis to assess the impact of *Mettl3* overexpression on myoblast differentiation. As shown in Fig. 3c–e, our findings revealed that following four days of differentiation, *Mettl3* overexpression significantly inhibits both the formation and differentiation of myoblasts, as well as their fusion. We conducted further transcriptomic sequencing and performed GO analysis (Fig. 3f, g, Supplementary Fig. 4a). Our results revealed that genes upregulated following *Mettl3* overexpression were significantly enriched in biological processes pertinent to the early stages of myoblast differentiation. For instance, the term “regulation of programmed cell death” specifically refers to apoptosis. Additionally, pathways such as “cell surface receptor signaling pathway”, “regulation of transmembrane receptor protein serine/threonine kinase signaling pathway”, and “cell surface receptor protein tyrosine kinase signaling pathway” are associated with cell fusion^24,25^. Genes that were down-regulated following *Mettl3* overexpression exhibited significant enrichment in biological processes during the later stages of differentiation, including “myofibril assembly”, “muscle contraction”, and “intracellular calcium ion homeostasis”. We also conducted a comprehensive analysis of the protein profiles in myoblasts. Notably, markers indicative of C2C12 myoblast differentiation, including myosin heavy chain 1 (MYH1), MYH3, and MYH7, exhibited significant upregulation during the differentiation process. However, these markers were markedly downregulated upon *Mettl3* overexpression (Supplementary Fig. 4b, c, Supplementary Data 4).

**Fig. 4:**
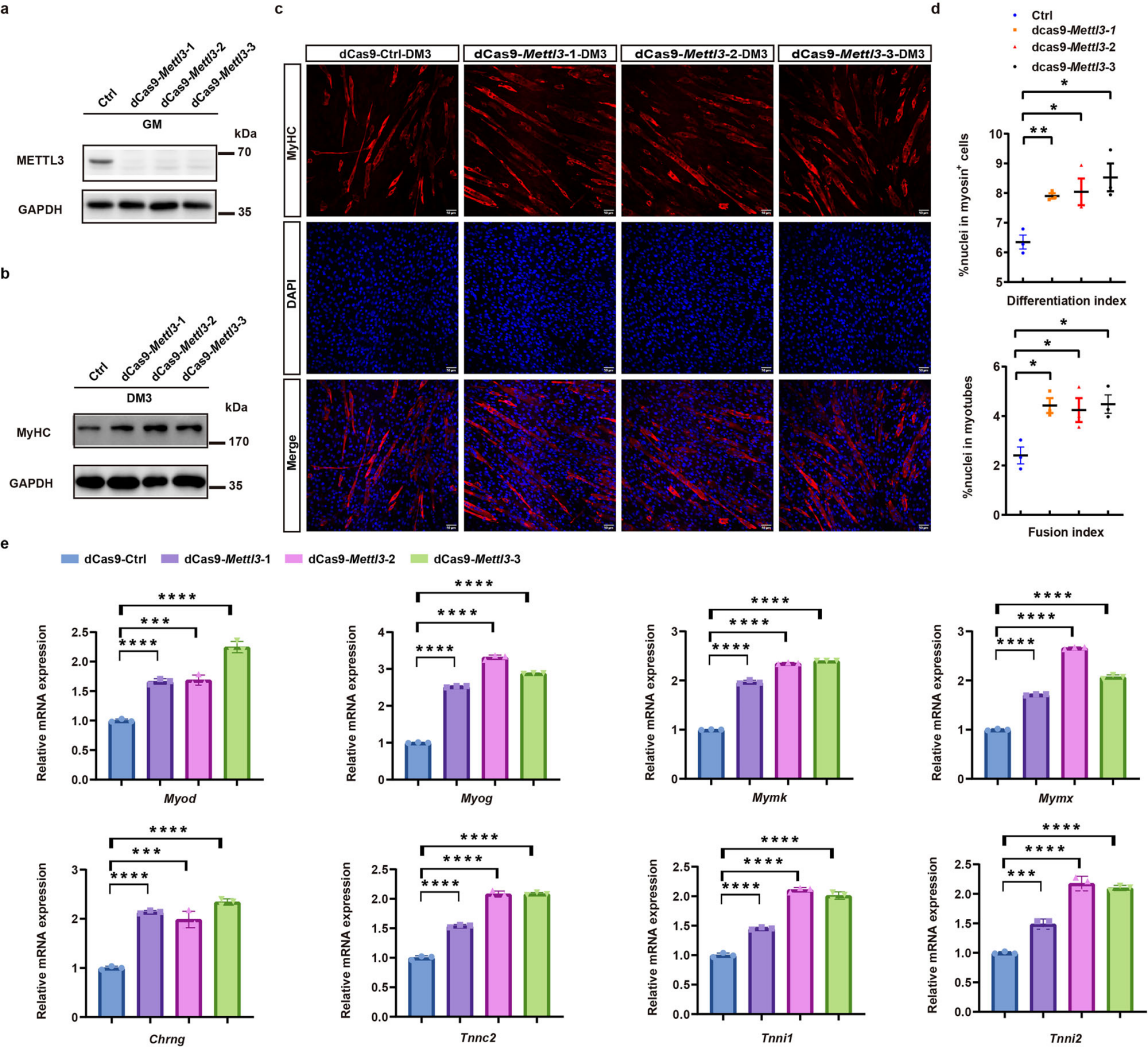
Inhibition of *Mettl3* expression affected the expression of genes related to myoblast fusion. **a** Immunoblotting analysis demonstrating METTL3 expression in *Mettl3* knockdown cells compared to the control group. **b** Immunoblotting analysis demonstrating MyHC expression in *Mettl3* knockdown cells compared to the control group. **c** Representative immunofluorescent staining of *Mettl3* knockdown cells compared to the control group on the third day post-differentiation (*n* = 3). Red indicated MyHC; blue indicated DAPI staining of nuclei. The merged images were shown. Scale bars, 50 μm. **d** Differentiation index was quantified from representative immunofluorescent images. The differentiation index is defined as the percentage of MyHC-positive cells relative to the total number of nuclei. The fusion index is defined as the ratio of the number of myotubes, characterized by MyHC-positive cells containing at least two nuclei, to the total number of nuclei in the field. **e** RT-qPCR analysis showing the expression levels of genes associated with myoblast fusion in *Mettl3* knockdown cells relative to the control group on the third day post-differentiation. Data presented as means ± SEM. ns. not significant, *P < 0.05, **P < 0.01, and ***P < 0.001, by two-sided Student’s *t* test, GAPDH was used as the internal control.

We subsequently employed dCas9 to suppress *Mettl3* expression in myoblasts (Fig. 4a). The results of the immunoblotting analysis demonstrated that inhibiting the expression of *Mettl3* could enhance the expression of MyHC, a key differentiation marker of myoblasts (Fig. 4b). We further conducted immunofluorescence analysis to evaluate the impact of *Mettl3* inhibition on myoblast differentiation (Fig. 4c). Our research findings indicate that the suppression of *Mettl3* expression significantly enhances the capacity of myoblasts to undergo fusion and form multinucleated myotubes (Fig. 4d). We further investigated the impact of *Mettl3* inhibition on the expression of genes associated with myoblast fusion and discovered that *Mettl3* inhibition substantially enhanced the expression levels of these genes following the induced differentiation of myoblasts (Fig. 4e). In contrast, prior to the induction of differentiation, *Mettl3* inhibition exhibited a slightly suppressive effect on the expression of certain genes, including *Myog* and *Mymk*, which are involved in myoblast fusion, as well as *Tnnc2*, *Tnni1*, and *Tnni2*, which play roles in myotube formation (Supplementary Fig. 4d). Following the induction and differentiation of myoblasts, the expression of *Mettl3* is significantly downregulated, while genes involved in regulating myoblast fusion and myotube formation begin to be expressed. This suggests that a high level of *Mettl3* expression acts as a barrier to the expression of these genes. Interestingly, prior to the induction and differentiation of myoblasts, the inhibition of high-level *Mettl3* expression suppresses the expression of genes involved in myoblast fusion and myotube formation. This suggests that these regulatory genes maintain a basal level of transcriptional activity before myoblast differentiation and are subsequently modulated by METTL3. However, it is only after the induction and differentiation of myoblasts that additional co-regulatory genes begin to express, at which point this gene group becomes functionally active and initiates its regulatory role.

Together, these results suggest that METTL3 is involved in the differentiation and fusion processes of myoblasts.

### m^6^A profiles in *Mettl3*-overexpressing myoblasts during differentiation

**Fig. 5:**
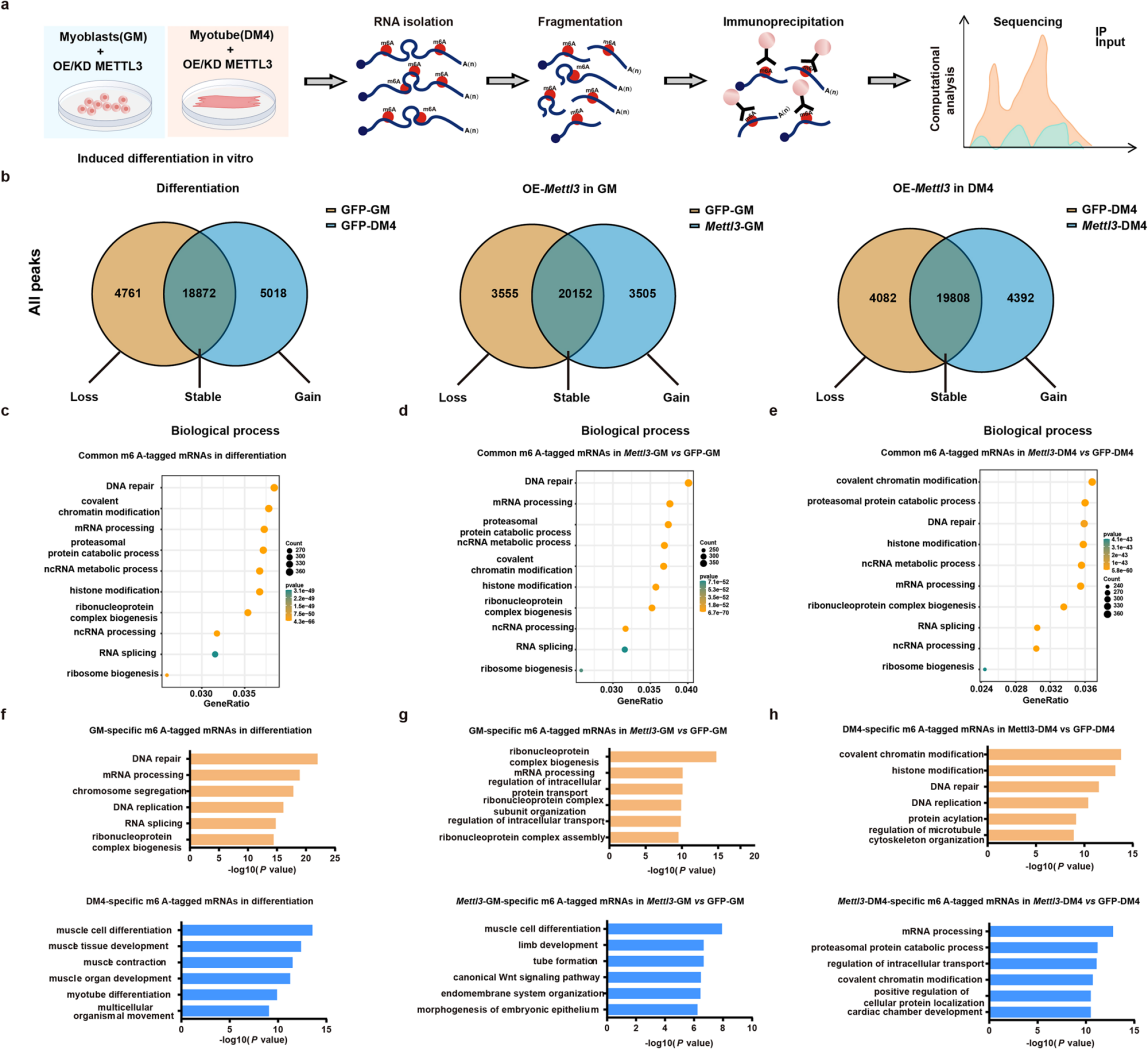
m^6^A profiles in *Mettl3*-overexpressing myoblasts during differentiation. **a** Schematic of the phenotypic analysis and m^6^A-seq procedure. **b** Venn diagram of peaks enriched in GFP-overexpressing cells and *Mettl3*-overexpressing cells. **c** GO analysis of genes encoding mRNAs with common m^6^As in GFP-overexpressing cells during differentiation. **d** GO analysis of genes encoding mRNAs with common m^6^As in *Mettl3*-overexpressing cells and GFP-overexpressing cells at GM stages. **e** GO analysis of genes encoding mRNAs with common m^6^As in *Mettl3*-overexpressing cells and GFP-overexpressing cells on the fourth day post-differentiation. **f** GO analysis of genes encoding mRNAs with specific m^6^As in GFP-overexpressing cells during differentiation. **g** GO analysis of genes encoding mRNAs with specific m^6^As in *Mettl3*-overexpressing cells and GFP-overexpressing cells prior to differentiation induction. **h** GO analysis of genes encoding mRNAs with specific m^6^As in *Mettl3*-overexpressing cells and GFP-overexpressing cells on the fourth day post-differentiation.

Given the robust functional roles of the methyltransferase METTL3 in m^6^A RNA modification, we performed m^6^A-seq to generate epitranscriptomic profiles, respectively (Fig. 5a). Prior to induction of differentiation (GM) and on the fourth day of induction (DM4), we characterized and compared myoblast samples overexpressing METTL3 with those from the control group overexpressing GFP. We conducted m^6^A methylation analysis to further elucidate the underlying mechanisms (Supplementary Fig. 5a, b, c). Additionally, the de novo motif predicted by HOMER revealed that the m^6^A sites of all the samples were highly concordant within a consensus motif (for example, the most enriched GGACU), with marked enrichment noted near stop codons (Supplementary Fig. 5d–g).

We identified more than 20,000 m^6^A peaks in each sample (Supplementary Data 5). To investigate potential differentiation-associated or METTL3-associated alterations in m^6^A epitranscriptomes, we categorized m^6^A peaks into three groups on the basis of their presence or absence fluctuations during differentiation or *Mettl3* overexpression. The majority of m^6^A peaks were consistently detected in both samples and were thus defined as stable m^6^A peaks. Conversely, a subset of m^6^A peaks exhibited specific detection patterns, which were classified as either loss or gain of m^6^A peaks (Fig. 5b, Supplementary Data 6). The majority of stable m^6^A peaks were consistently observed during differentiation or *Mettl3* overexpression, whereas the presence and absence of m^6^A peaks exhibited significant variability (Supplementary Data 7, 8). Functional pathway enrichment analysis of genes for which their mRNAs harbored common m^6^A peaks revealed high enrichment in terms of a variety of essential functions, such as covalent chromatin modification, DNA repair and RNA metabolism-related processes (Fig. 5c-e, Supplementary Fig. 6, Supplementary Fig. 7 and Supplementary Data 9). During differentiation, genes encoding mRNAs with specific m^6^A peaks demonstrated high enrichment in terms of myogenesis-associated functions, such as muscle cell differentiation, muscle tissue development and muscle contraction (Fig. 5f). In the context of *Mettl3* overexpression prior to differentiation induction, genes encoding mRNAs with specific m^6^A peaks were highly enriched in functions related to myogenesis and embryonic development, such as muscle cell differentiation, limb development, tube formation and the wnt signaling pathway (Fig. 5g). After sustaining *Mettl3* overexpression during the DM4 phase, genes encoding mRNAs with specific m^6^A peaks exhibited significant enrichment in functions related to proteasomal protein catabolic processes, mRNA processing and intracellular transport (Fig. 5h). Furthermore, we conducted a functional analysis of RNAs exhibiting altered m^6^A peaks in *Mettl3* knockdown cells to comprehensively evaluate the functional impact of METTL3 on myoblast differentiation and myogenesis (Supplementary Fig. 8). To elucidate the relationship between RNA methylation dynamics and gene expression alterations through METTL3 regulation during differentiation, we investigated the expression level and divergence of mRNAs characterized by loss, gain, and stable m^6^A peaks in *Mettl3*-overexpressing or inhibition myoblasts compared with the control group (Supplementary Fig. 9, Supplementary Fig. 10).

Collectively, these findings indicate that m^6^A peaks are prevalent throughout the process of differentiation and display a remarkable level of specificity associated with this process. However, the m^6^A peak modified by METTL3 exhibits notable differences before and after induced differentiation. In addition to regulating essential functions, m^6^A may also contribute to myoblast differentiation-specific alterations during myogenesis.

### Features of METTL3-regulated lncRNA m^6^A alterations during differentiation

**Fig. 6:**
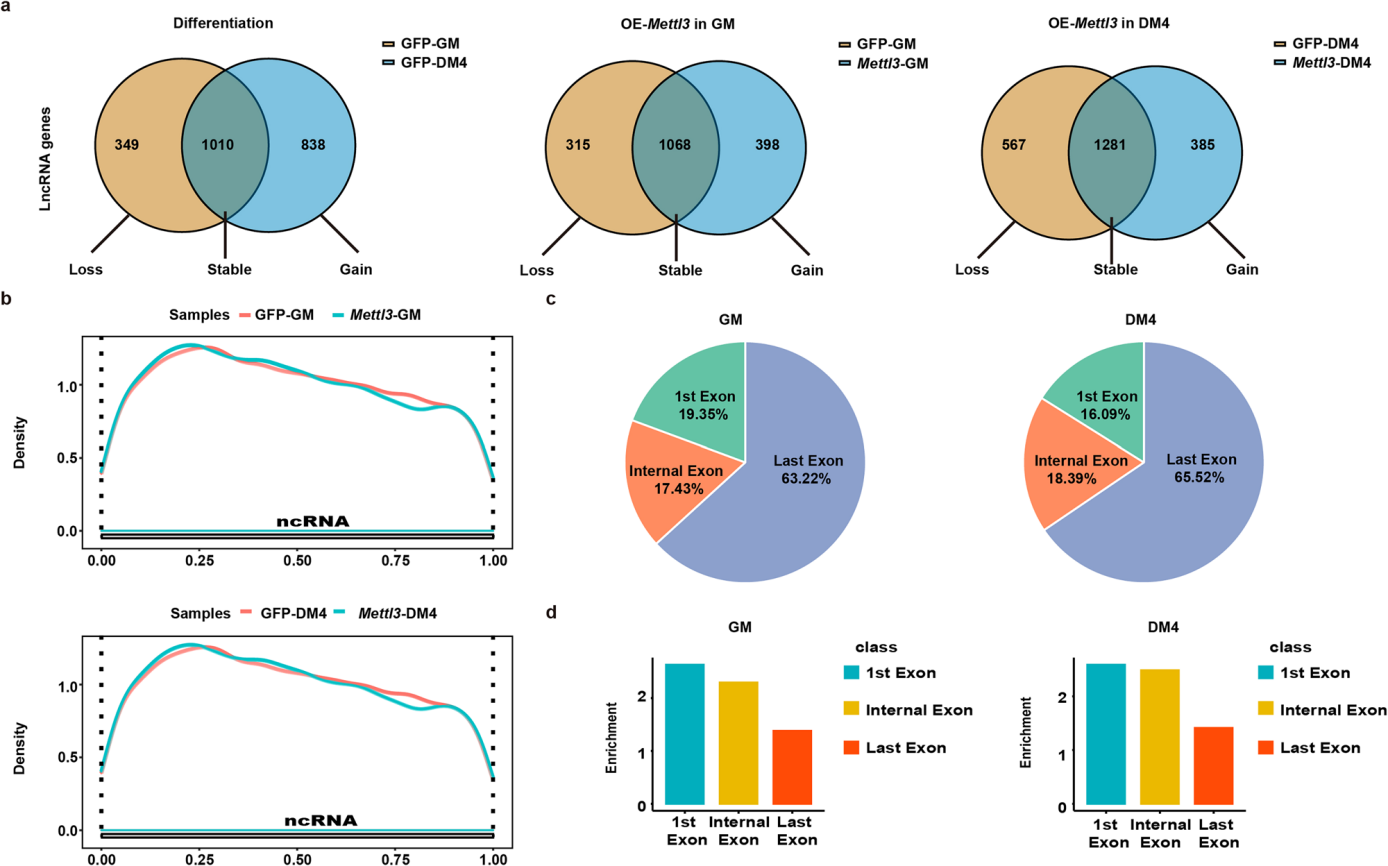
Features of METTL3 regulated lncRNA m^6^A alterations during differentiation. **a** Venn diagram of lncRNA peaks enriched in GFP-overexpressing cells and *Mettl3*-overexpressing cells. **b** Metagene profiles of enrichment of all m^6^A peaks across lncRNAs transcriptome in *Mettl3*-overexpressing cells and GFP-overexpressing cells before and after induced differentiation. **c** Pie charts represent the proportion of m^6^A peaks in the three regions of lncRNAs before and after induced differentiation. **d** Histogram represents the relative enrichment of m^6^A peaks in the three regions of lncRNAs before and after induced differentiation.

Given the altered m^6^A modification and lncRNA expression profiles during myoblast differentiation^26^, we further investigated the m^6^A methylation landscape of long non-coding RNAs (lncRNAs) (Fig. 6a, Supplementary Fig.11). Surprisingly, the *Mettl3* overexpression-mediated losses in lncRNA m^6^A peaks exhibited a significant increase at the DM4 stage, which contrasts with the statistical results for the mRNAs shown in Fig. 5. We explored the distribution of peaks along the lncRNA gene body and found that its density gradually decreased from the transcription initiation site to the transcriptional termination site (Fig. 6b). We further analyzed the peak distribution of the lncRNA exons. We found that m^6^A peaks were preferentially enriched in the last exons of lncRNAs expressed in *Mettl3*-overexpressing samples. In total, 63.22% and 65.52% of the m^6^A peaks were identified in the last exons of the lncRNAs expressed before and after induced differentiation, respectively (Fig. 6c). We then analyzed the peak enrichment in each lncRNA. Interestingly, we found that m^6^A peaks were preferentially enriched in the first and internal exon but not in the last exons (Fig. 6d). These data provide a fundamental reference for further studies of the m^6^A epitranscriptome. Subsequently, we conducted an analysis of the differential expression of m^6^A-modified lncRNAs during myoblast differentiation and in *Mettl3*-overexpressing cells (Supplementary Data 10). We subsequently performed Venn diagram analysis to examine the overlap of DEGs in myoblasts during differentiation, in cells overexpressing *Mettl3* prior to differentiation induction, and in cells overexpressing *Mettl3* on the fourth day post-differentiation (Supplementary Data 11). A total of five identical lncRNAs were identified across all three groups. In addition to these five RNAs, 38 genes exhibited differential expression between myoblasts during differentiation and METTL3-overexpressing cells at the DM4 stage. We screened this set of genes and selected those that showed opposite trends and significant changes for literature review (Supplementary Fig. 11a, b). According to existing literature, several lncRNAs play significant roles in the musculoskeletal system. Notably, 2310043L19Rik is highly expressed in muscle tissue, functioning as a competing endogenous RNA (ceRNA) that targets miR-125a-5p. This interaction promotes myoblast proliferation while inhibiting their differentiation^27^. NEAT1 has been identified as dysregulated in various neuromuscular disorders^28^. Specifically, its expression is downregulated in murine models of muscular dystrophy and markedly upregulated in amyotrophic lateral sclerosis (ALS)^29^. BC1 may play a role in the prenatal development and differentiation of muscle tissues^30^. Gm5532 exerts its activatory effect on osteoclast differentiation through modulation of the miR-125a-3p/TRAF6 axis^31^. Furthermore, several reported lncRNAs, including Gm2694 and AI480526, have been implicated in the pathogenesis of neurological disorders, particularly depression^32,33^.

### METTL3 regulates skeletal muscle fusion in an m^6^A-dependent manner

**Fig. 7:**
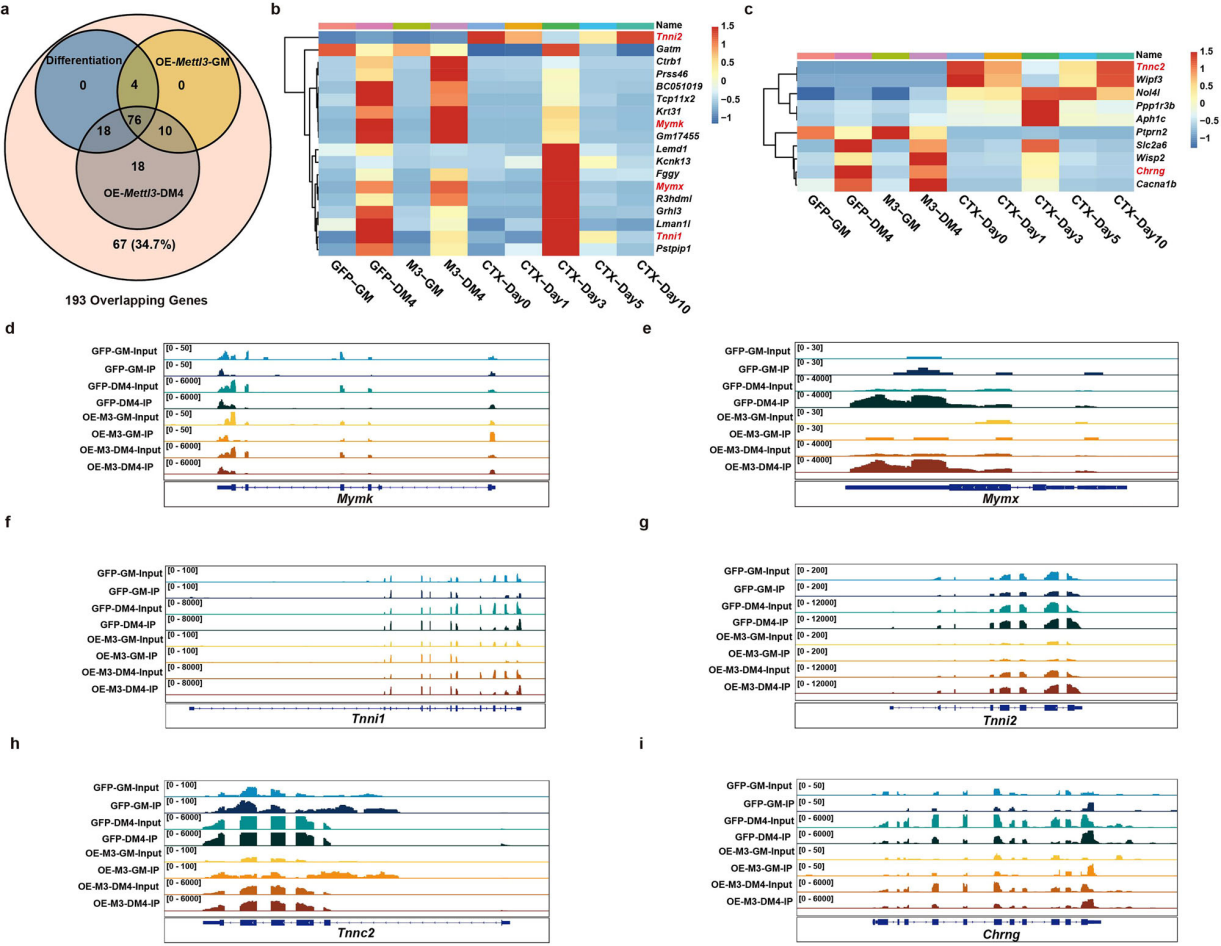
METTL3 regulates skeletal muscle fusion in an m^6^A-dependent manner. **a** Venn diagram showing the number of overlapping DEGs between the four comparison groups during skeletal muscle regeneration (big circles) and the number of differentially expressed transcripts that also contain m^6^As in *Mettl3*-overexpressing cells and GFP-overexpressing cells before and after induced differentiation (small circles). **b** Heatmap of differentially expressed transcripts with m^6^As in *Mettl3*-overexpressing cells on the fourth day post-differentiation. **c** Heatmap of differentially expressed transcripts with m^6^As in *Mettl3*-overexpressing cells prior to differentiation induction. **d** Integrative Genomics View (IGV) of input and immunoprecipitation overlays on the *Mymk* gene from the MeRIP-seq data set for *Mettl3*-overexpressing cells and GFP-overexpressing cells before and after induced differentiation. **e** IGV of input and immunoprecipitation overlays on the *Mymx* gene from the MeRIP-seq data set for *Mettl3*-overexpressing cells and GFP-overexpressing cells before and after induced differentiation. **f** IGV of input and immunoprecipitation overlays on the *Tnni1* gene from the MeRIP-seq data set for *Mettl3*-overexpressing cells and GFP-overexpressing cells before and after induced differentiation. **g** IGV of input and immunoprecipitation overlays on the *Tnni2* gene from the MeRIP-seq data set for *Mettl3*-overexpressing cells and GFP-overexpressing cells before and after induced differentiation. **h** IGV of input and immunoprecipitation overlays on the *Tnnc2* gene from the MeRIP-seq data set for *Mettl3*-overexpressing cells and GFP-overexpressing cells before and after induced differentiation. **i** IGV of input and immunoprecipitation overlays on the *Chrng* gene from the MeRIP-seq data set for *Mettl3*-overexpressing cells and GFP-overexpressing cells before and after induced differentiation.

Bioinformatic cross-analysis of m^6^A-mediated myogenesis-associated mRNAs from the 193 overlapping mRNAs involved in skeletal muscle regeneration against all the m^6^A-containing mRNAs previously identified by meRIP-seq revealed that ~65.3% of the mRNAs that underwent regeneration-associated differential expression were also m^6^A-containing mRNAs (Fig. 7a). The analysis revealed that among the 193 DEGs, 126 were found to contain m^6^A modifications. Specifically, 98 DEGs were enriched in the differentiation process, 90 DEGs were enriched in cells overexpressing *Mettl3* prior to differentiation induction, and an additional 122 DEGs were enriched in cells overexpressing *Mettl3* on the fourth day post-differentiation (Supplementary Data 12). The samples overexpressing *Mettl3* before the induction of differentiation showed modification of 10 mRNAs via METTL3-regulated m^6^A. In contrast, on the fourth day after differentiation, 18 mRNAs in these samples were modified by METTL3-regulated m^6^A. We subsequently analyzed the expression of enriched genes with mRNAs that included METTL3-regulated m^6^As across different samples (Supplementary Fig. 13). Genes encoding mRNAs with METTL3-regulated m^6^As were enriched in terms of myoblast fusion (for example, *Mymk*, *Mymx*, and *Chrng*) and skeletal muscle contraction (for example, *Tnni1*, *Tnni2* and *Tnnc2*) (Fig. 7b, c)^24,34,35^.

**Fig. 8:**
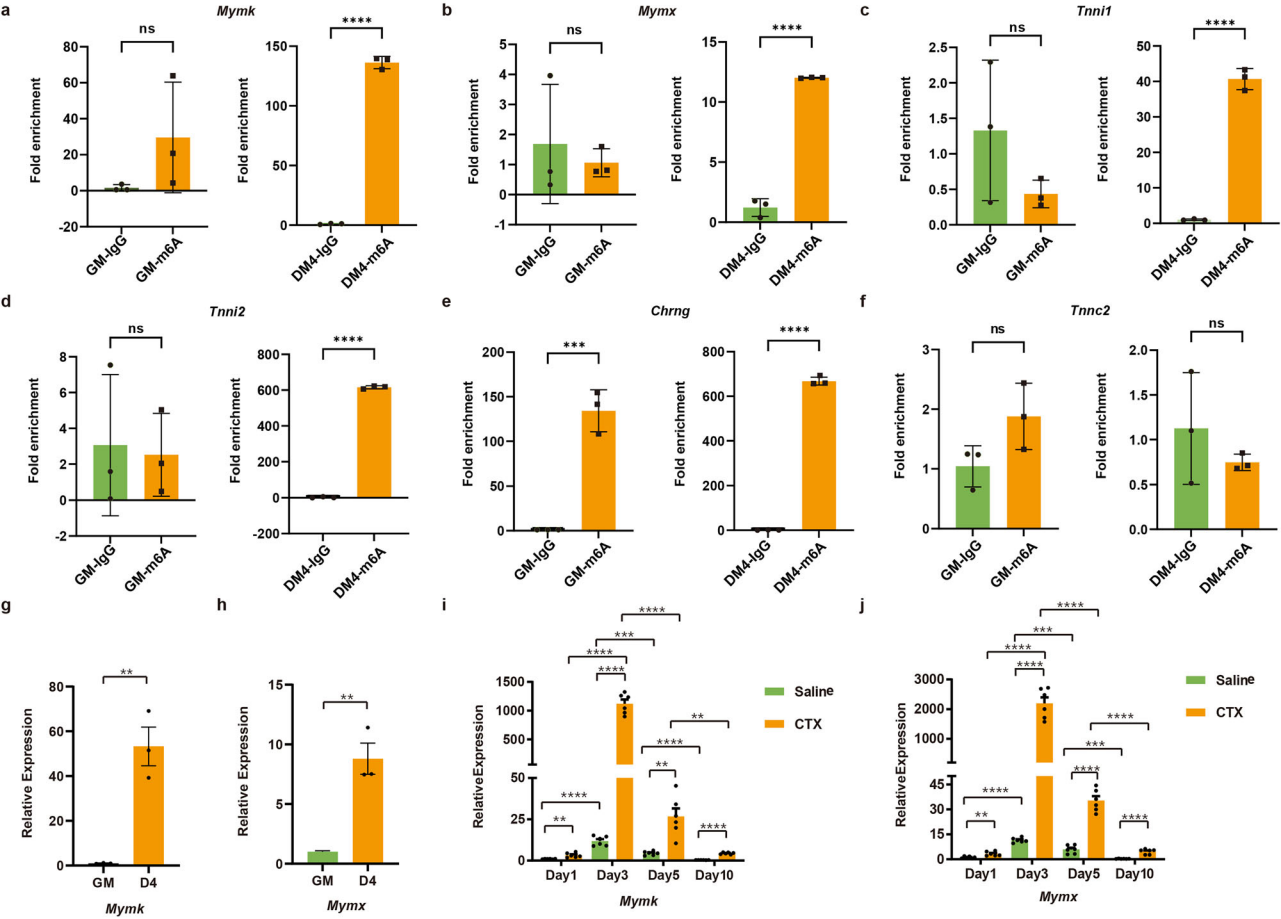
Methylated RIP–qPCR analysis to examine m^6^A and expression levels of mRNAs with METTL3-regulated m^6^As. **a** RT-qPCR analysis of *Mymk* expression in immunoprecipitated RNAs during differentiation. **b** RT-qPCR analysis of *Mymx* expression in immunoprecipitated RNAs during differentiation. **c** RT-qPCR analysis of *Tnni1* expression in immunoprecipitated RNAs during differentiation. **d** RT-qPCR analysis of *Tnni2* expression in immunoprecipitated RNAs during differentiation. **e** RT-qPCR analysis of *Chrng* expression in immunoprecipitated RNAs during differentiation. **f** RT-qPCR analysis of *Tnnc2* expression in immunoprecipitated RNAs during differentiation. IgG Immunoprecipitation was used as negative control. **g** RT-qPCR analysis of *Mymk* expression during myoblasts differentiation. **h** RT-qPCR analysis of *Mymx* expression during myoblasts differentiation. **i** RT-qPCR analysis of *Mymk* expression from TA muscles at 1-, 3-, 5- and 10-days following CTX-induced skeletal muscle injury. **j** RT-qPCR analysis of *Mymx* expression from TA muscles at 1-, 3-, 5- and 10-days following CTX-induced skeletal muscle injury. Data presented as means ± SEM. ns. not significant, *P < 0.05, **P < 0.01, and ***P < 0.001, by two-sided Student’s *t* test.

We subsequently analyzed the m^6^A peaks of these mRNAs and identified METTL3-regulated m^6^As across different samples. Our meRIP-seq data revealed one clear m^6^A peak around the 3ʹUTRs of *Mymk*, *Mymx*, *Chrng*, *Tnni1*, *Tnni2* and *Tnnc2* mRNAs during differentiation (Fig. 8d–i). To confirm that these mRNAs were enriched in m^6^A marks, we used an antibody against m^6^A and performed RNA immunoprecipitation followed by real-time PCR. As shown in Fig. 8, compared with those in the IgG control group, these mRNAs were significantly enriched in the m^6^A group at the DM4 stage (Fig. 8a–f). During the process of normal skeletal muscle differentiation, a gradual downregulation in *Mettl3* expression was observed, whereas m^6^A-modified *Mymk* and *Mymx* showed significant upregulation of expression upon induction of differentiation and regeneration (Fig. 8g–j). Our findings suggested that aberrant overexpression of *Mettl3* following skeletal muscle differentiation resulted in a reduction in the m^6^A modification levels of *Mymk* and *Mymx*. Taken together, these findings suggest that many mRNAs undergo m^6^A modification with minimal alterations in their RNA expression levels. Notably, the fusion factors *Mymk* and *Mymx* were identified as m^6^A-enriched transcripts involved in skeletal muscle differentiation.

## Discussion

Skeletal muscle, the most abundant tissue and protein reservoir, not only governs movement but also plays a pivotal role in regulating respiration, ingestion, energy expenditure, glucose metabolism, amino acid utilization, lipid homeostasis, and maintenance of an optimal quality of life^36–38^. Impairments in muscle function can lead to a spectrum of adverse outcomes, including compromised ambulation, impaired respiratory function and ultimately premature mortality^21^. Precise regulation is imperative for skeletal muscle regeneration processes. The molecular regulatory mechanisms governing skeletal muscle differentiation and regeneration constitute the primary focus of research on skeletal muscle function. However, a significant gap in our understanding of the apparent involvement of RNA in this process remains. To date, more than 100 types of RNA modifications have been identified, with methylation emerging as the predominant form of modification. The expression of METTL3 and its m^6^A modifications are significantly differentially regulated during skeletal muscle regeneration^39^. However, the current study highlights the lack of systematic investigations into how METTL3 is involved in regulating skeletal muscle regeneration; thus, it is crucial to elucidate the mechanism underlying the role of METTL3 and develop precise epigenetic therapeutic strategies.

In our study, we characterized the involvement of METTL3 in the m^6^A-modified transcriptome during myoblast differentiation. We observed significant upregulation of METTL3 following CTX-induced skeletal muscle injury and identified numerous alterations in myogenic pathways and genes associated with *Mettl3* expression. Additionally, distinct patterns of gene-specific m^6^A modification were observed during skeletal muscle differentiation, with some genes exhibiting loss of m^6^As and others showing an increase in m^6^As. Importantly, we discovered that METTL3 serves as a key regulator responsible for these gene-specific m^6^A modifications. Notably, downstream elements regulated by METTL3, such as *Mymx* and *Mymk*, which are involved in myoblast fusion, presented increased expression levels and m^6^A levels during muscle formation. This study provides valuable insights into the function and mechanism of METTL3-regulated m^6^A modifications in myoblast differentiation and skeletal muscle regeneration, revealing the regulatory role of METTL3 in promoting myoblast fusion, a crucial step in myogenesis^40,41^. Furthermore, our analysis successfully identified a subset of long noncoding RNAs within this group of genes. Currently, there is a dearth of systematic research on the involvement of m^6^A-mediated lncRNA methylation in the regulation of myogenic differentiation and regeneration, necessitating further elucidation of the regulatory mode and mechanism^42^. Therefore, elucidating the mode of lncRNA-m^6^A methylation in skeletal muscle and exploring its regulatory mechanism are highly important for understanding the role of m^6^A-modified lncRNAs in body development and regeneration while expanding our understanding of the involvement of m^6^A modification in cell differentiation and tissue repair.

Mounting evidence indicates the involvement of m^6^A regulation in cellular senescence and aging-related processes, underscoring its potential as a therapeutic target for aging^43,44^. In this context, METTL3-catalyzed m^6^A modification enhances mRNA stability through IGF2BP1 recruitment^45^. In primate aging, diminished *Mettl3* expression and overall RNA m^6^A levels are associated with skeletal muscle degradation^11^. Disruptions in m^6^A patterns have also been associated with age-related diseases such as Alzheimer’s disease, osteoarthritis, and disc degeneration^46,47^. These findings underscore the importance of m^6^A and its regulators in processes related to cellular aging and regeneration. Therefore, a systematic analysis of the regulatory network governing m^6^A during the regeneration process may aid in elucidating its underlying mechanism.

## Materials and methods

### Animals

C57BL/6 J mice were purchased from Cyagen and housed in temperature-controlled, humidity-controlled, and ventilated specific pathogen-free cages at the animal facility. All animal handling and procedures were approved by the Animal Care and Use Committee at South China Agricultural University (permission no. 2023F257). For the mouse muscle injury and regeneration experiments, the tibialis anterior (TA) muscles of six-week-old male mice were injected with 25 μL of 10 μM cardiotoxin (CTX, Merck Millipore, 217503), and 0.9% normal saline was used as a control. The regenerated muscles were collected at days 1, 3, 5 and 10 postinjection. TA muscles were isolated for hematoxylin and eosin (H&E) staining or frozen in liquid nitrogen for RNA and protein extraction.

### Cell cultures

The mouse skeletal myoblast cell line C2C12 was obtained from the Shanghai Institute of Cell Biology, Chinese Academy of Science. The cells were cultured in growth medium (GM), which consisted of high-glucose Dulbecco’s modified Eagle’s medium (DMEM, Thermo Fisher) supplemented with 10% FBS (Thermo Fisher) and 1% penicillin/streptomycin (Thermo Fisher), at 37 °C in 5% CO_2_. The cells were plated and cultured to 100% confluence and then transferred to differentiation medium consisting of DMEM containing 2% horse serum (Thermo Fisher) and 1% penicillin/streptomycin for further culture. Transfection of plasmid DNA was performed using ViaFect™ Transfection Reagent (Promega) according to the manufacturer’s instructions.

### Plasmids construction and stable cell generation

cDNAs of mouse METTL3 were subcloned and inserted into the pKD-CMV-MCS-EF1-PURO (pKD) vector by Gibson assembly, and pKD-GFP was subcloned as a negative control. To establish METTL3 knockdown using a CRISPR-dCas9 system, two complementary single-guide RNA (sgRNA) sequences targeting the METTL3 promoter region were first cloned into the lentiviral vector pLV hU6-sgRNA hUbC-dCas9-KRAB-T2a-Puro (Addgene #71236) via BsmBⅠ restriction enzyme digestion and Golden Gate assembly. The vector features an hU6 promoter for sgRNA transcription, an hUbC promoter driving expression of a catalytically inactive dCas9 (D10A/H840A mutations) fused to the Krüppel-associated box (KRAB) transcriptional repressor domain at its C-terminus, and a T2A self-cleaving peptide-linked puromycin resistance gene (Puro^R^).

For lentiviral production, HEK-293T cells were co-transfected with the sgRNA-dCas9-KRAB transfer vector, psPAX2 (encoding HIV-1 gag/pol for viral core assembly), and pMD2.G (expressing VSV-G envelope glycoprotein for broad tropism) at a molar ratio of 4:3:1 using polyethylenimine (PEI). Virus-containing supernatant was harvested 48 h post-transfection, filtered through a 0.45-μm PVDF membrane, and stored at –80 °C. C2C12 cells were infected with lentiviral particles at a multiplicity of infection of puromycin selection (2 μg/mL) was initiated 72 h post-infection and maintained for 2 days to generate stable cell lines. All primer sequences are listed in Supplementary Table1.

### RNA extraction and qPCR assays

Total RNA was extracted from mouse muscles or C2C12 cells with TRIzol reagent (Invitrogen) according to the manufacturer’s instructions. First-strand cDNA for PCR analyses was synthesized with a PrimeScript™ RT reagent kit (Takara). Real-time PCR was performed using SYBR Premix ExTaq™ (Takara) in a sequence detection system (Thermo Fisher). The Ct values were first normalized to those of the endogenous control (GAPDH) and then normalized to those of the control group (ΔΔCT method) to calculate the fold change between the control and experimental groups. All primer sets were synthesized by Synbio Technologies, and all primer sequences are listed in Supplementary Table2.

### Protein isolation and immunoblotting analysis

The tissue was homogenized and then lysed in ice-cold RIPA buffer (50 mM Tris-HCl (pH 7.4), 150 mM NaCl, 0.1% SDS, 1% sodium deoxycholate, 1% Triton X-100, 2 mM EDTA and 1× protease inhibitor cocktail). The samples were then centrifuged for 20 min at 4 °C. Total protein extracts were loaded and separated using SDS‒polyacrylamide gel electrophoresis (SDS‒PAGE) and then transferred to nitrocellulose membranes (Whatman). The membranes were then blocked with 5% milk for 1 h. The membranes were incubated with primary antibodies against MyHC (R&D Cat. MAB4470), METTL3 (15073-1-AP), and GAPDH (CST Cat. 2118 T) overnight at 4 °C. Horseradish peroxidase-conjugated secondary antibodies were used to detect the primary antibodies, and protein signals were then visualized using a chemiluminescent HRP substrate (Millipore, WBKLS0500).

### Immunofluorescence

Cells were fixed with 4% formaldehyde for 30 min at room temperature, followed by permeabilization using 0.1% Triton X-100 for 10 min on ice. Next, cells were blocked with 2.5% bovine serum albumin (BSA) in phosphate-buffered saline (PBS; Beyotime) for 2.5 h at room temperature. After blocking, the cells were incubated overnight at 4 °C with a primary antibody against the myosin heavy chain (MyHC). The cells were subsequently washed three times with PBS for 10 min each and incubated with an Alexa Fluor 647-conjugated secondary antibody (Invitrogen) for 1 h at room temperature. Nuclei were stained with DAPI (Invitrogen) for 10 min. Fluorescence signals were captured and analyzed using laser scanning confocal microscopy (Olympus).

### Immunocytochemistry (IHC)

The tissue sections were cut at a thickness of 4 μm and then subjected to a water temperature increase of 42 °C followed by oven baking at 60 °C for 30 min. The samples were dewaxed with xylene I for 5 min, xylene II for 5 min, xylene III for 5 min, anhydrous ethanol for 1 min, 95% ethanol for 1 min, 75% ethanol for 1 min, and finally washed with distilled water for 5 min. EDTA microwave hot repair was carried out for 5–8 min, followed by cooling to room temperature. To prevent reagent flow-out, an immunohistochemical pen circle was used. Endogenous peroxidase blocking solution was added, and the samples were incubated at room temperature for 10 min before being washed with PBS three times. Sealing serum was added, and the mixture was incubated at 37 °C for 30 min without washing away the excess serum. Primary antibody incubation involved the addition of primary antibody dropwise, followed by wet box incubation at 37 °C for 2 h. The samples were incubated with secondary antibodies at 37  °C for 30 min before being washed with PBS three times. DAB color development solution consisting of DAB:1 mL B solution + 1 drop of solution A mixed together was added to the samples. Mayer hematoxylin was added briefly, followed by washing with distilled water and soaking in blue liquid for one minute before another wash. Dehydration and transparent sealing involved gradient dehydration using alcohol concentrations ranging from 75–95–100%, with each concentration lasting one minute per cylinder.

### RNA m^6^A quantification by LC-MS/MS

RNA m^6^A quantification by LC-MS/MS was performed by metware Corporation. Briefly, total RNAs were isolated using TRIzol reagent (Life Technologies). 1ug mRNA was incubated with nuclease S1 (Takara), phosphodiesterase (Sigma-Aldrich) and alkaline phophatase (Takara), and incubated at 37 °C for 2 h. Following centrifugation at 13,000 rpm for 10 min at 4 °C, 10 μL of the solution was analyzed by LC-MS/MS.

### MeRIP sequencing

Total RNA was isolated and purified using TRIzol reagent (Invitrogen) following the manufacturer’s procedure. The RNA amount and purity of each sample were quantified via a NanoDrop ND-1000 (NanoDrop). The RNA integrity was assessed with a Bioanalyzer 2100 (Agilent) with a RIN > 7.0 and confirmed by electrophoresis with a denaturing agarose gel. Poly(A) RNA was purified from 50 µg of total RNA using Dynabeads Oligo (dT) 25-61005 (Thermo Fisher Scientific). Then, the poly(A) RNA was fragmented into small pieces via the Magnesium RNA Fragmentation Module (NEB). Then, the cleaved RNA fragments were incubated with an m^6^A-specific antibody (#202003, Synaptic Systems, Germany). Then, the IP RNA was reverse transcribed to create cDNA by SuperScriptTM II Reverse Transcriptase (Invitrogen, cat. 1896649), which was subsequently fled to synthesize U-labeled second-strand DNAs. After ligation with the adapter to the A-tailed fragmented DNA, the ligated products were amplified with PCR, and 2 × 150 bp paired-end sequencing (PE150) was performed on an Illumina NovaSeqTM 6000 (LC-Biotechnology Co., Ltd., Hangzhou, China) following the vendor’s recommended protocol.

### RNA sequencing and analysis

RNA sequencing was performed by Epigenome Corporation. Briefly, total RNA or purified sRNA fragments of the samples were extracted and first ligated to the 3’-terminal and 5’-terminal linkers and then reverse-transcribed into cDNA. PCR amplification was performed, and the gel was then cut to recover the target fragment library. In silico sequencing was performed on libraries that passed the quality inspection.

### Statistics and reproducibility

For the meRIP-seq analysis, we employed fastp software to filter out reads containing adaptor contamination, low-quality bases, and undetermined bases using default parameters. Subsequently, the sequence quality of both IP and input samples was assessed with fastp. HISAT2 was utilized to align the reads to the reference genome (GRCm38, Ensembl), retaining uniquely mapped reads with a mapping quality score exceeding 30^48^. The aligned reads from IP and input libraries were then analyzed using the R package exomePeak for identifying significant m^6^A peaks and differential peaks, considering an FDR ≤ 0.05 as statistically significant^49^. For visualization purposes, the BigWig format files were generated using deepTools and visualized with IGV software^50,51^. HOMER was applied for de novo motif discovery based on the top 1000 most enriched peaks^52^. Peaks were annotated by intersecting them with gene architecture using bedtools and custom Python scripts^53^. An m^6^A metagene plot was created using the Guitar package in R^54^.

For mRNA-seq analysis, clean FASTQ reads were aligned to the mouse genome (Ensembl GRCm38) using HISAT2. Raw gene read counts were calculated using featureCounts based on Ensembl gene annotation. These counts were normalized to RPKM values using the fpkm function in the DESeq2 package^55^. were identified via DESeq2, with genes having an adjusted *P* value ≤ 0.05 and logFC ≥2 considered statistically significant. Gene Ontology and KEGG pathway analyses were conducted using the clusterProfiler package, with *p* values ≤ 0.05 deemed statistically significant^56^. Heatmaps and Circos plots were generated using Graphbio1 software (http://www.graphbio1.com/).

The data is presented as means with standard errors of the mean (SEMs) from three independent experiments, unless otherwise specified in the figure legends. In each graph, individual dots represent single biological replicates, and *P* values are indicated. Each experiment is conducted using a minimum of three independent biological samples, as detailed in the figure legends. Comparisons between two groups were analyzed using Student’s *t* test, unless otherwise stated. All statistical analyses and graphical representations were performed using GraphPad Prism software (version 9.0). Results were considered statistically significant when *P* < 0.05.

The schematic diagrams in Figs. 1a,  5a were created using PowerPoint software and Adobe Illustrator CS6. The mouse illustration in Fig. 1a was adapted from the article titled “Genome-wide identification of microRNA targets reveals positive regulation of the Hippo pathway by miR-122 during liver development,” published by one of our manuscript’s authors, Tan, in the journal *Cell Death & Disease* in 2021.

### Reporting summary

Further information on research design is available in the Nature Portfolio Reporting Summary linked to this article.

## Supplementary information

Supplementary Information

Dataset 1

Dataset 2

Dataset 3

Dataset 4

Dataset 5

Dataset 6

Dataset 7

Dataset 8

Dataset 9

Dataset 10

Dataset 11

Dataset 12

Reporting Summary

Description of Additional Supplementary Files

## Data Availability

All data needed to evaluate the conclusions in the paper are presented in the paper and/or the Supplementary Data Set. Unedited Western blots are included in Supplementary Figs. 14, 15, and 16 in Supplementinuteary Information. The meRIP-seq data and RNA sequencing data that support the findings of this study have been deposited in the Gene Expression Omnibus (GEO) under accession codes GSE293208 and GSE293212. All other data supporting the findings of this study are available from the corresponding author upon request.
