# Supplementary material for: Stage-specific requirement for METTL3-dependent m6A epitranscriptomic regulation during myogenesis
Source: Commun Biol. 2025 Aug 30;8:1317. doi: 10.1038/s42003-025-08759-5 (PMC12398539; doi:10.1038/s42003-025-08759-5)

**SUPPLEMENTARY INFORMATION**

**Related to Tan et al. "Stage-specific requirement for METTL3-dependent m6A epitranscriptomic regulation during myogenesis"**

**Supplementary Table 1. Primer sequences of plasmids construction**

| | sequence (5'-->3') |
|---|---|
| pKD-mMettl3-CEII-F | tccaggcctaagcttacgcgtATGTCGGACACGTGGAGCTC |
| pKD-mMettl3-CEII-R | gtcgtccttgtagtcgaattcTAAATTCTTAGGTTTAGAGATGATGCCG |
| | sequence (5'-->3') |
| dCas9-METTL3-1-F | CACCGAGCTAGGATGTCGGACACG |
| dCas9-METTL3-1-R | CGTGTCCGACATCCTAGCTCGGTG |
| dCas9-METTL3-2-F | caccCGGAAAGGGCGCAGAGCCCG |
| dCas9-METTL3-2-R | aaacCGGGCTCTGCGCCCTTTCCG |
| dCas9-METTL3-3-F | caccGATGGGGTAACCAACAATCG |
| dCas9-METTL3-3-R | aaacCGATTGTTGGTTACCCCATC |

**Supplementary Table 2. Primer sequences of qPCR assays**

| | |
|---|---|
| m-Tnni1-F | ATGCCGGAAGTTGAGAGGAAA |
| m-Tnni1-R | TCCGAGAGGTAACGCACCTT |
| m-Tnni2-F | AGAGTGTGATGCTCCAGATAGC |
| m-Tnni2-R | AGCAACGTCGATCTTCGCA |
| m-Tnnc2-F | GAGGCCAGGTCCTACCTCAG |
| m-Tnnc2-R | GGTGCCCAACTCTTTAACGCT |
| m-Chrng-F | GGCCAGAGACCTCATCTCCT |
| m-Chrng-R | GGGGTCGTAGTTTCGCATCA |
| m-MYMK-F | ACTTTTCATGACCGCTGGG |
| m-MYMK-R | ATGCTCTTGTCGGGGTACAG |
| m-MYMX-F | TCCCAAGACATGAGAGAGGC |
| m-MYMX-R | TACTTTGATGGGCGTTGCTG |

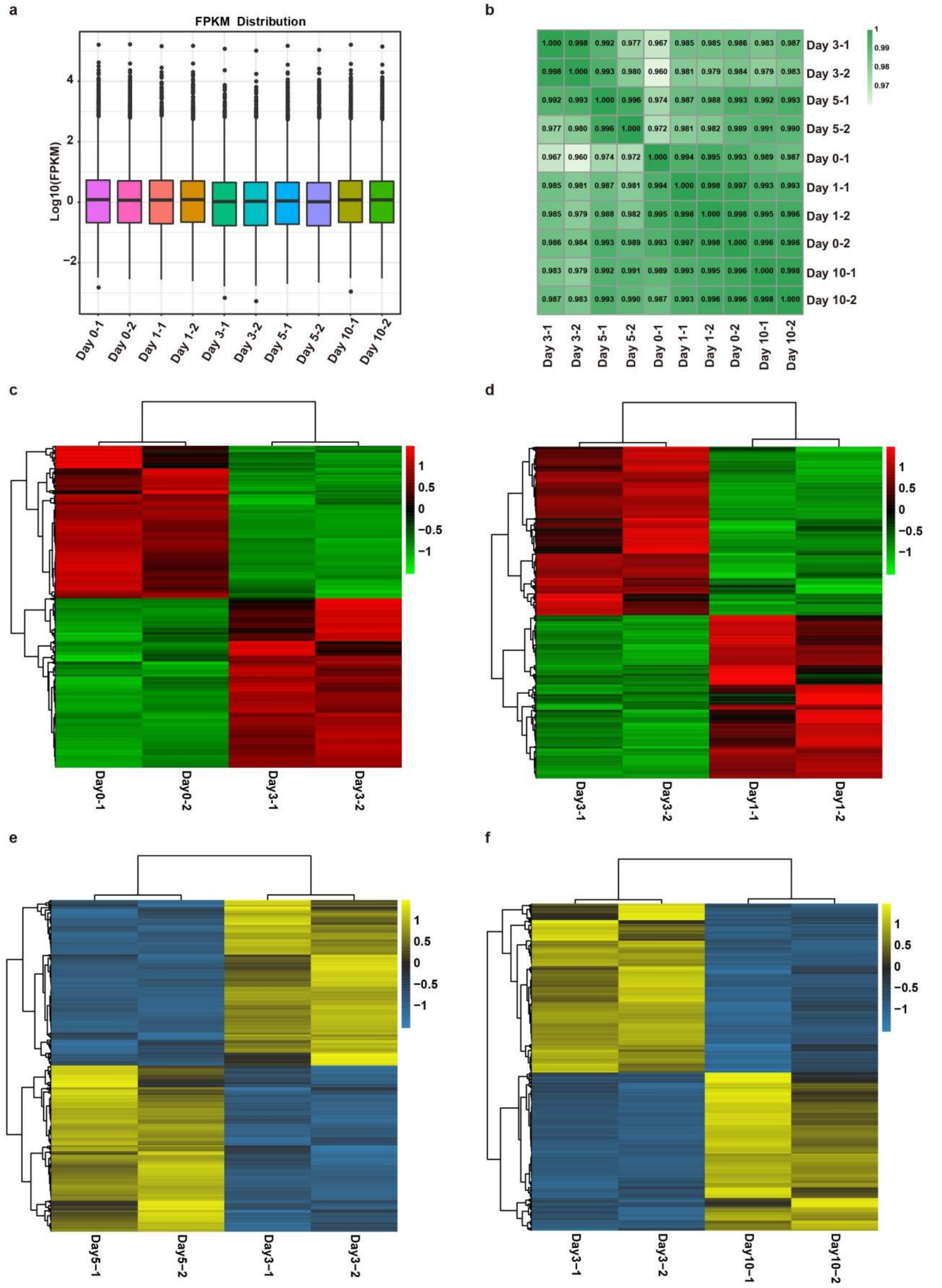


**Supplementary Fig 1. Identification and integrative analysis of DEGs during skeletal muscle regeneration. a** Violin plot of expression distribution in all samples during skeletal muscle regeneration. **b** Correlation between all samples during skeletal muscle regeneration. **c** Heatmap illustrates the gene expression between the third day

post-injury and pre-injury conditions. **d** Heatmap illustrateshe gene expression between the first- and third-day post-injury. **e** Heatmap illustrates he gene expression between day 3 and day 5 post-injury. **f** Heatmap illustrates he gene expression between day 3 and day 10 post-injury.

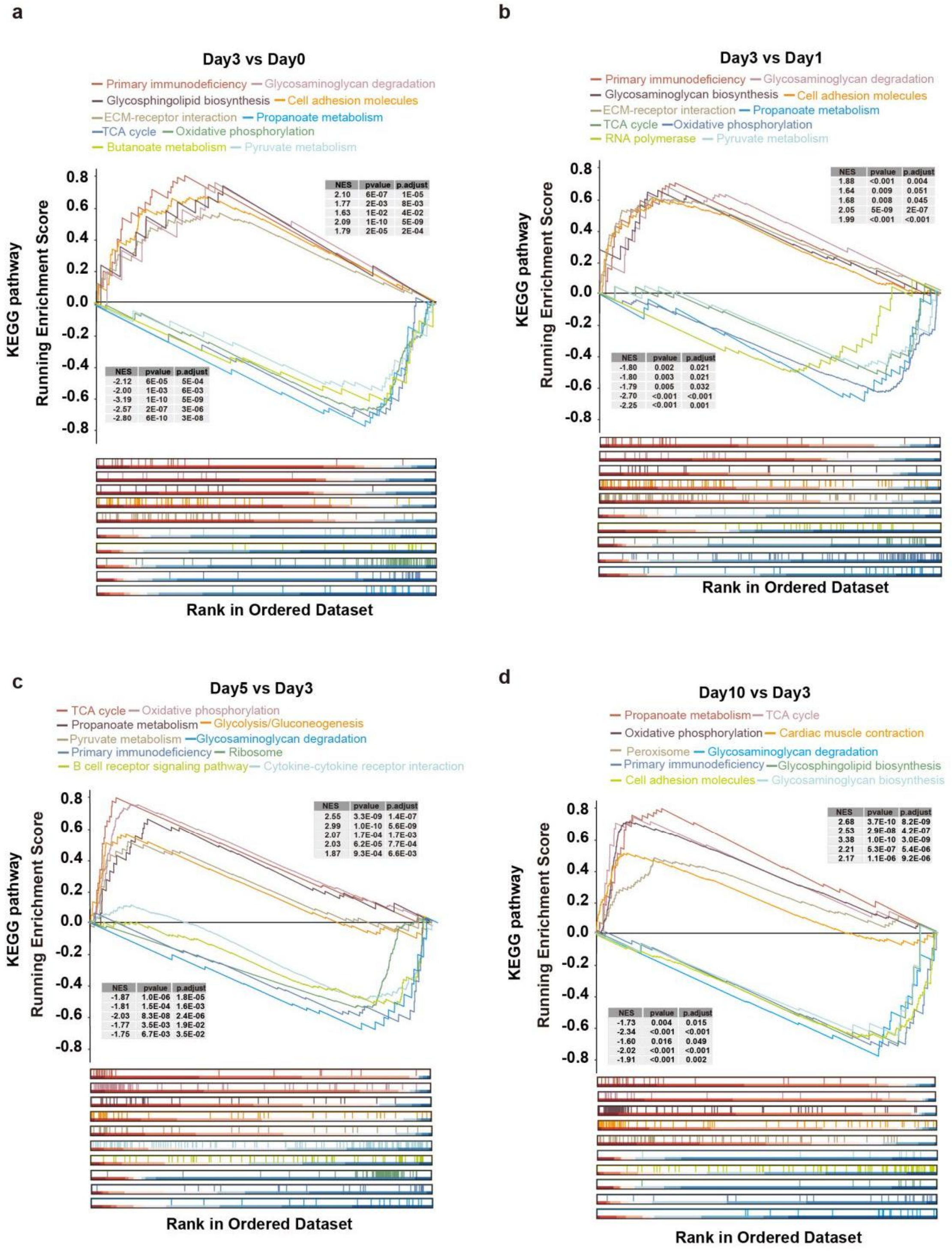


**Supplementary Fig 2. Gene set enrichment analysis (GSEA) of KEGG pathway.** **a** GSEA was performed on the entire set of DEGs between the third day post-injury

and pre-injury conditions. **b** GSEA was performed on the entire set of DEGs between the first- and third-day post-injury. **c** GSEA was performed on the entire set of DEGs between day 3 and day 5 post-injury. **d** GSEA was performed on the entire set of DEGs between day 3 and day 10 post-injury.

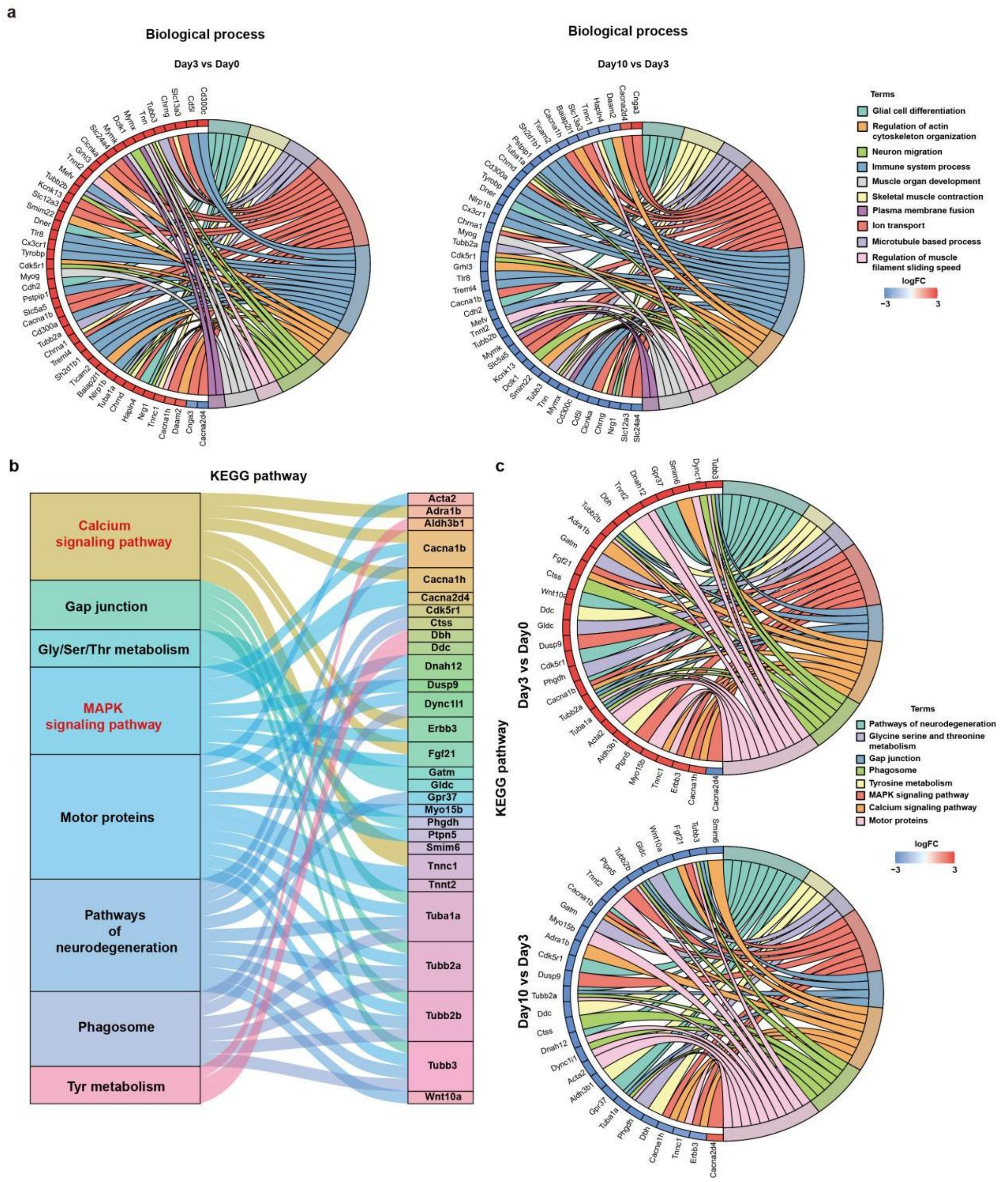


**Supplementary Fig 3. Analysis of gene expression during skeletal muscle regeneration.** **a** Chord diagram shows the expression changes of TOP genes in this group of enrichment biological process at different time points. **b** Sankey map illustrates the KEGG pathway of TOP genes enrichment. **c** Chord diagram shows the

expression changes of TOP genes in this group of enrichment KEGG pathways at different time points.

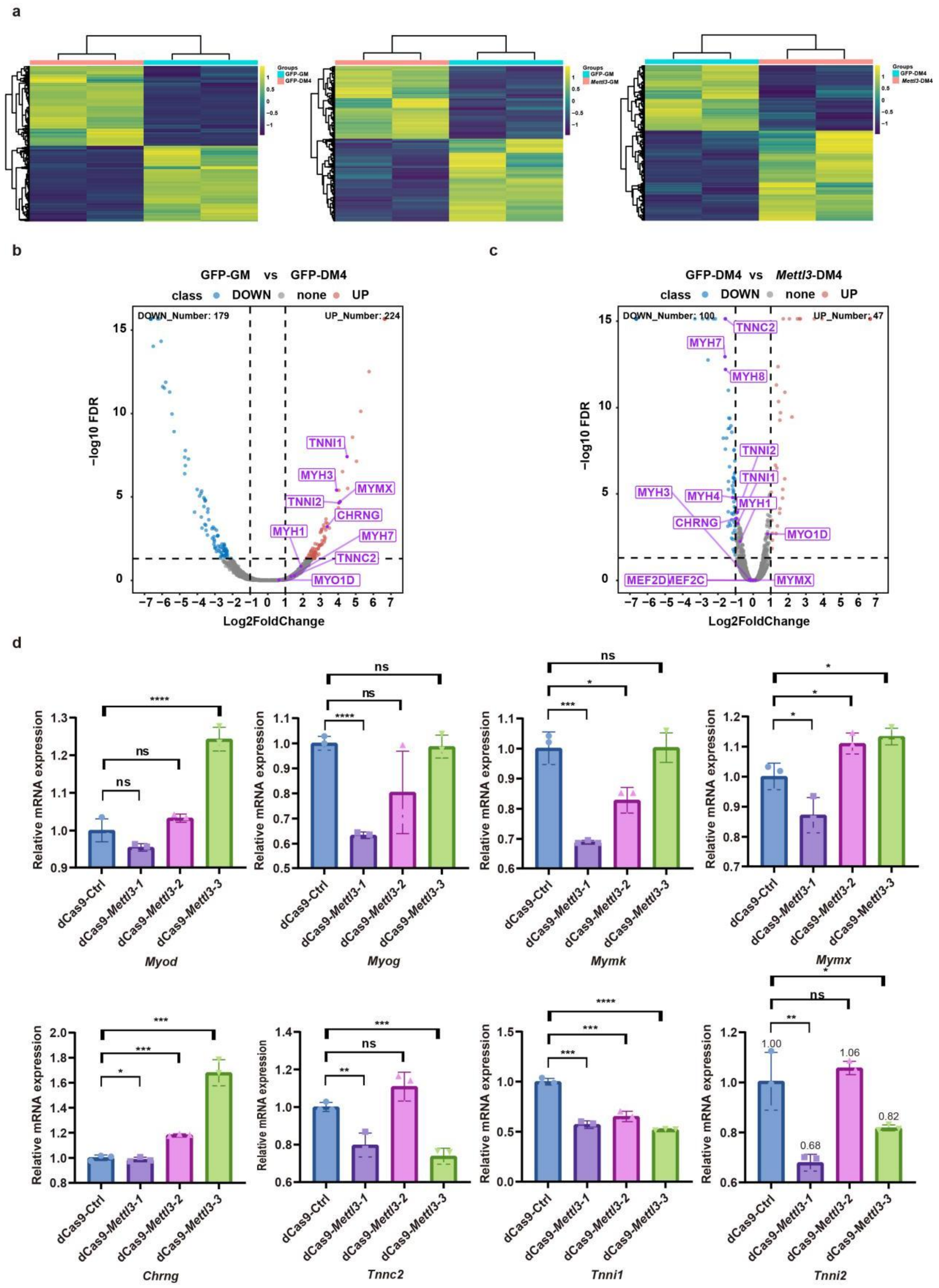


**Supplementary Fig 4. Analysis of the role of METTL3 in myoblast differentiation and fusion processes. a** Heatmap illustrates the gene expression in *Mettl3*-overexpressing cells and GFP-overexpressing cells during differentiation. **b**

Proteomic profiling of DEGs in GFP-overexpressing cells during differentiation. **c** Proteomic profiling of *Mettl3*-overexpressing cells compared to the control group on the fourth day post-differentiation. **d** RT-qPCR analysis showing the expression levels of genes associated with myoblast fusion in Mettl3 knockdown cells relative to the control group prior to differentiation induction. Data presented as means ± SEM. ns., not significant, $*P < 0.05$, $**P < 0.01$, and $***P < 0.001$, by two-sided Student's t-test.

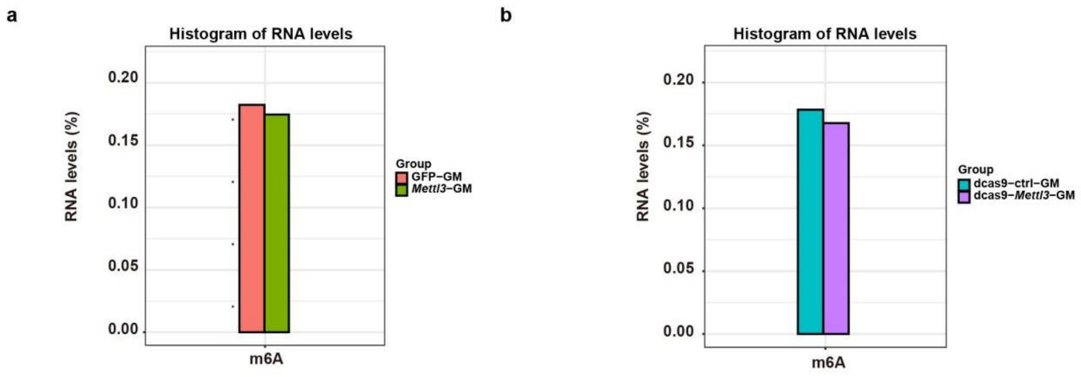


| Quantitative analysis of m6A levels | | | |
|---|---|---|---|
| GFP−GM | *Mettl3*−GM | dcas9−ctrl−GM | dcas9−*Mettl3*−GM |
| 0.182361 | 0.174553 | 0.17838 | 0.167665 |

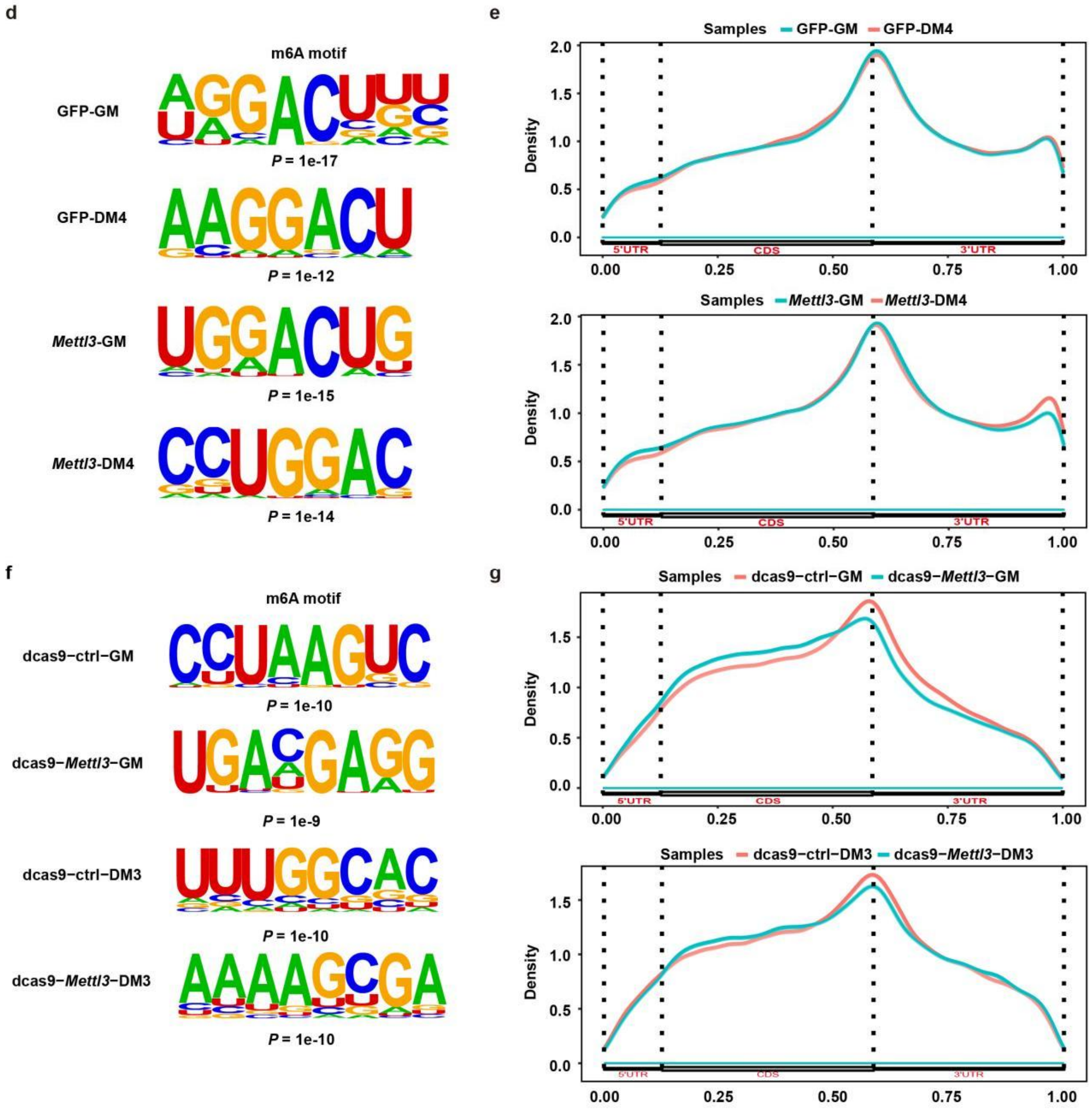


**Supplementary Fig 5. m$^6$A profiles in *Mettl3*-overexpressing and *Mettl3* knockdown myoblasts during differentiation.** **a** Quantitative analysis of m$^6$A levels in *Mettl3*-overexpressing cells compared to the control group. **b** Quantitative analysis

of $m^6A$ levels in *Mettl3* knockdown cells compared to the control group. **c** Table of quantitative analysis of $m^6A$ levels. **d** Top consensus motif identified with $m^6A$ peaks in *Mettl3*-overexpressing cells and wildtype cells from. **e** Peaks detected in samples were plotted via density across mRNA regions (including 5'UTR, start codon, CDS, stop codon, and 3' UTR). **f** Top consensus motif identified with $m^6A$ peaks in *Mettl3* knockdown cells and the control group. **g** Peaks detected in samples were plotted via density across mRNA regions (including 5'UTR, start codon, CDS, stop codon, and 3' UTR).

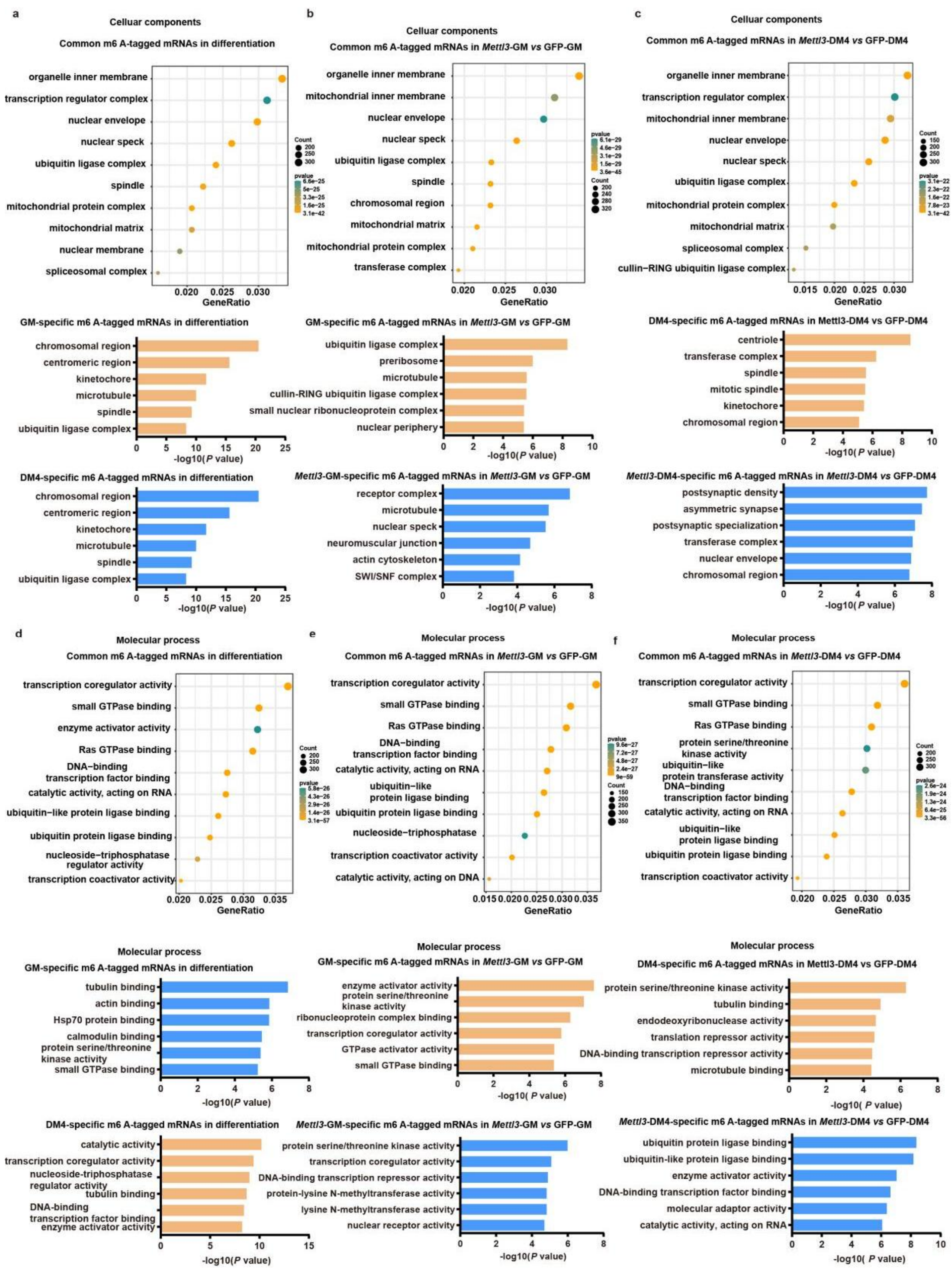


**Supplementary Fig 6. GO analysis of genes encoding mRNAs with common m$^6$As and specific m$^6$As. a** GO analysis (cellular components) of genes encoding mRNAs with common m$^6$As and specific m$^6$As in GFP-overexpressing cells during differentiation. **b** GO analysis (cellular components) of genes encoding mRNAs with common m$^6$As and specific m$^6$As in *Mettl3*-overexpressing cells and GFP-overexpressing cells prior to differentiation induction. **c** GO analysis (cellular

components) of genes encoding mRNAs with common m$^6$As and specific m$^6$As in *Mettl3*-overexpressing cells and GFP-overexpressing cells on the fourth day post-differentiation. **d** GO analysis (molecular process) of genes encoding mRNAs with common m$^6$As and specific m$^6$As in GFP-overexpressing cells during differentiation. **e** GO analysis (molecular process) of genes encoding mRNAs with common m$^6$As and specific m$^6$As in *Mettl3*-overexpressing cells and GFP-overexpressing cells prior to differentiation induction. **f** GO analysis (molecular process) of genes encoding mRNAs with common m$^6$As and specific m$^6$As in *Mettl3*-overexpressing cells and GFP-overexpressing cells on the fourth day post-differentiation.

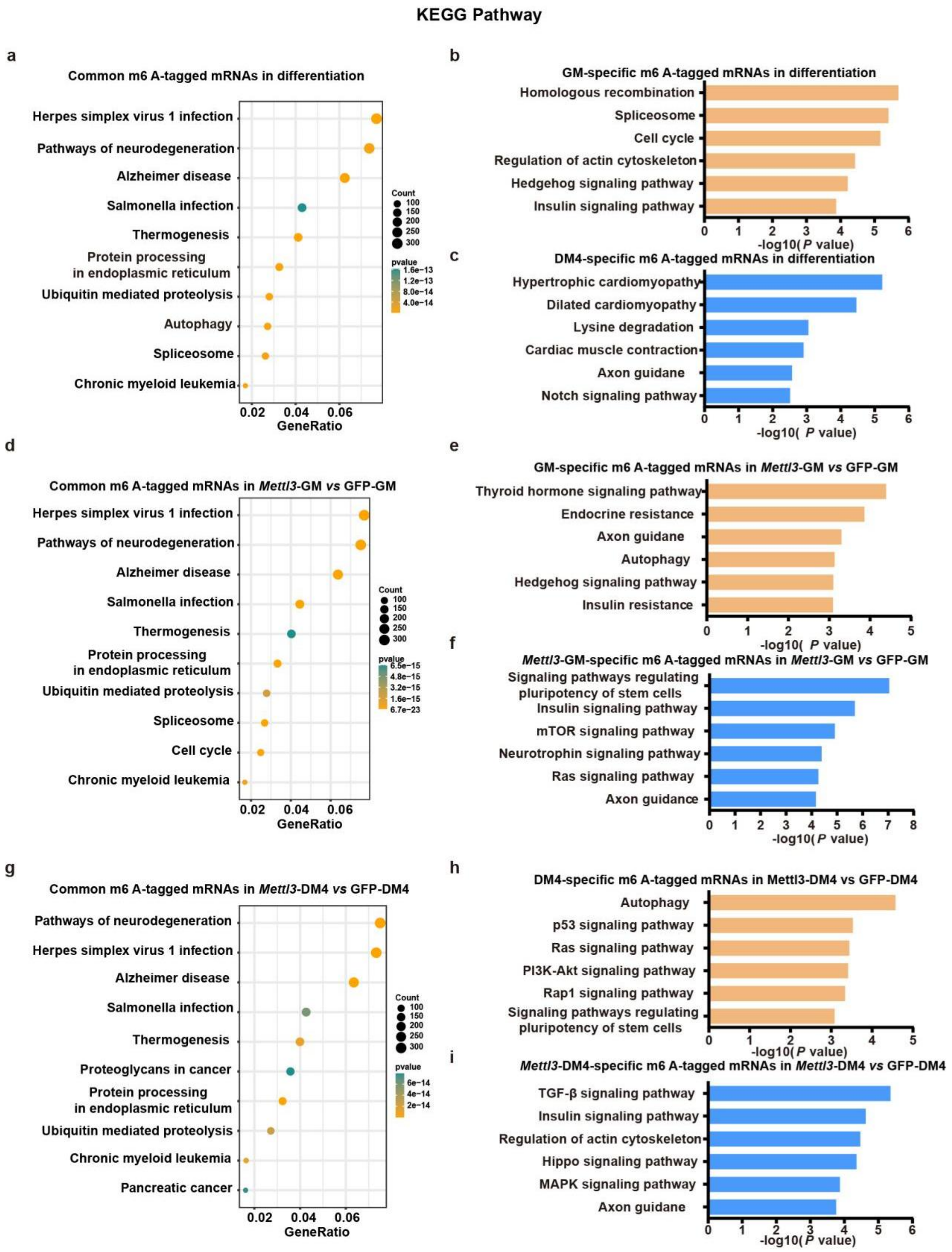


**Supplementary Fig 7. KEGG analysis of genes encoding mRNAs with common m$^6$As and specific m$^6$As. a** KEGG analysis of genes encoding mRNAs with common m$^6$As in GFP-overexpressing cells during differentiation. **b** KEGG analysis of genes encoding mRNAs with specific m$^6$As in GFP-overexpressing cells prior to differentiation induction. **c** KEGG analysis of genes encoding mRNAs with specific m$^6$As in GFP-overexpressing cells on the fourth day post-differentiation. **d** KEGG

analysis of genes encoding mRNAs with common m$^6$As in *Mettl3*-overexpressing cells and GFP-overexpressing cells prior to differentiation induction. **e** KEGG analysis of genes encoding mRNAs with specific m$^6$As in GFP-overexpressing cells prior to differentiation induction. **f** KEGG analysis of genes encoding mRNAs with specific m$^6$As in *Mettl3*-overexpressing cells prior to differentiation induction. **g** KEGG analysis of genes encoding mRNAs with common m$^6$As in *Mettl3*-overexpressing cells and GFP-overexpressing cells on the fourth day post-differentiation. **h** KEGG analysis of genes encoding mRNAs with specific m$^6$As in *Mettl3*-overexpressing cells on the fourth day post-differentiation. **i** KEGG analysis of genes encoding mRNAs with specific m$^6$As in *Mettl3*-overexpressing cells on the fourth day post-differentiation.

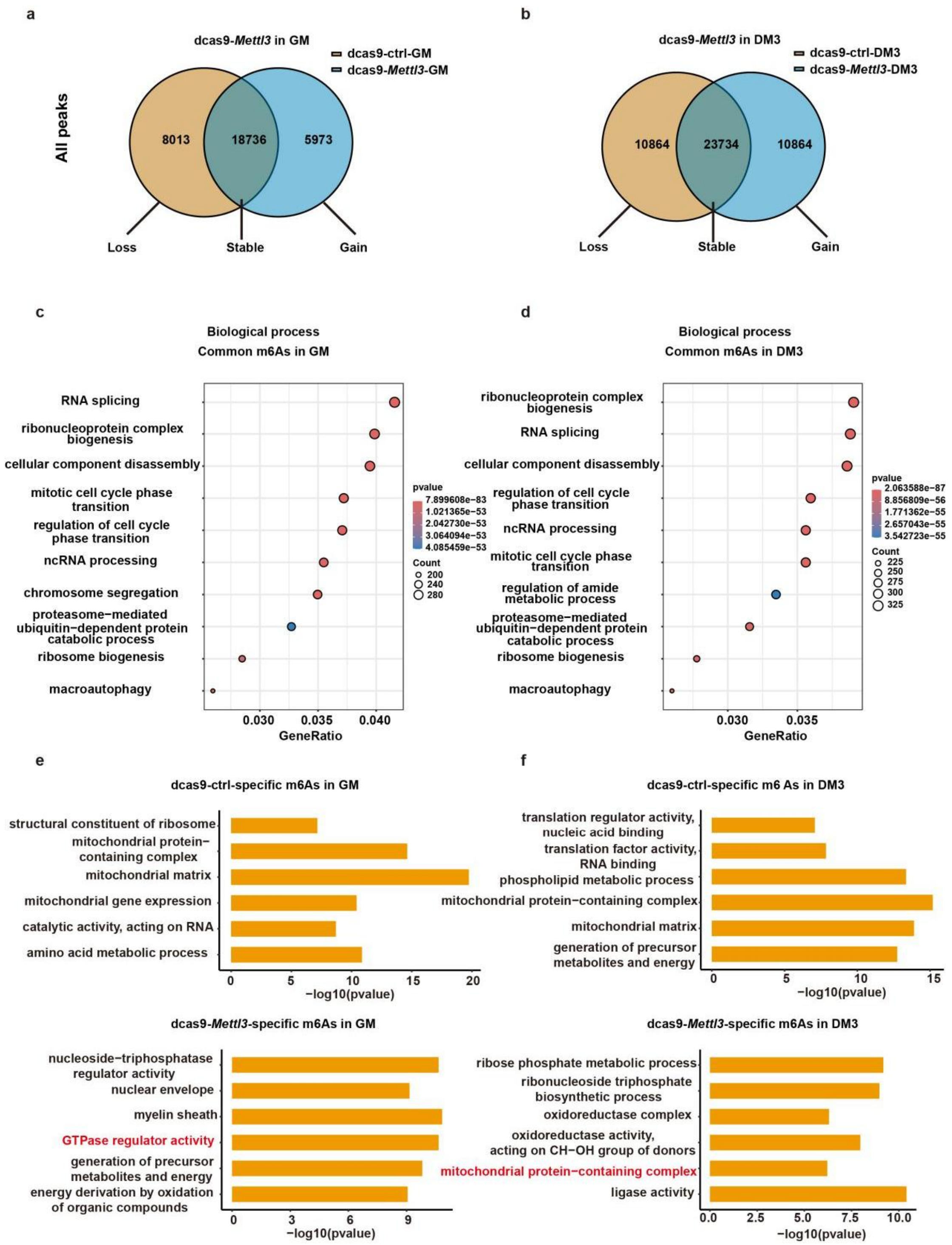


**Supplementary Fig 8. METTL3 regualted m$^6$A alterations in *Mettl3* knockdown myoblasts during differentiation.** **a** Venn diagram illustrating the overlap of peaks enriched in *Mettl3* knockdown cells compared to the control group prior to differentiation induction. **b** Venn diagram illustrating the overlap of peaks enriched in *Mettl3* knockdown cells compared to the control group on the third day post-differentiation. **c** GO analysis of genes encoding mRNAs with common m$^6$As in

*Mettl3* knockdown cells compared to the control group prior to differentiation induction. **d** GO analysis of genes encoding mRNAs with common m$^6$As in in *Mettl3* knockdown cells compared to the control group on the third day post-differentiation. **e** GO analysis of genes encoding mRNAs with specific m$^6$As in *Mettl3* knockdown cells compared to the control group prior to differentiation induction. **f** GO analysis of genes encoding mRNAs with specific m$^6$As in *Mettl3* knockdown cells compared to the control group on the third day post-differentiation.

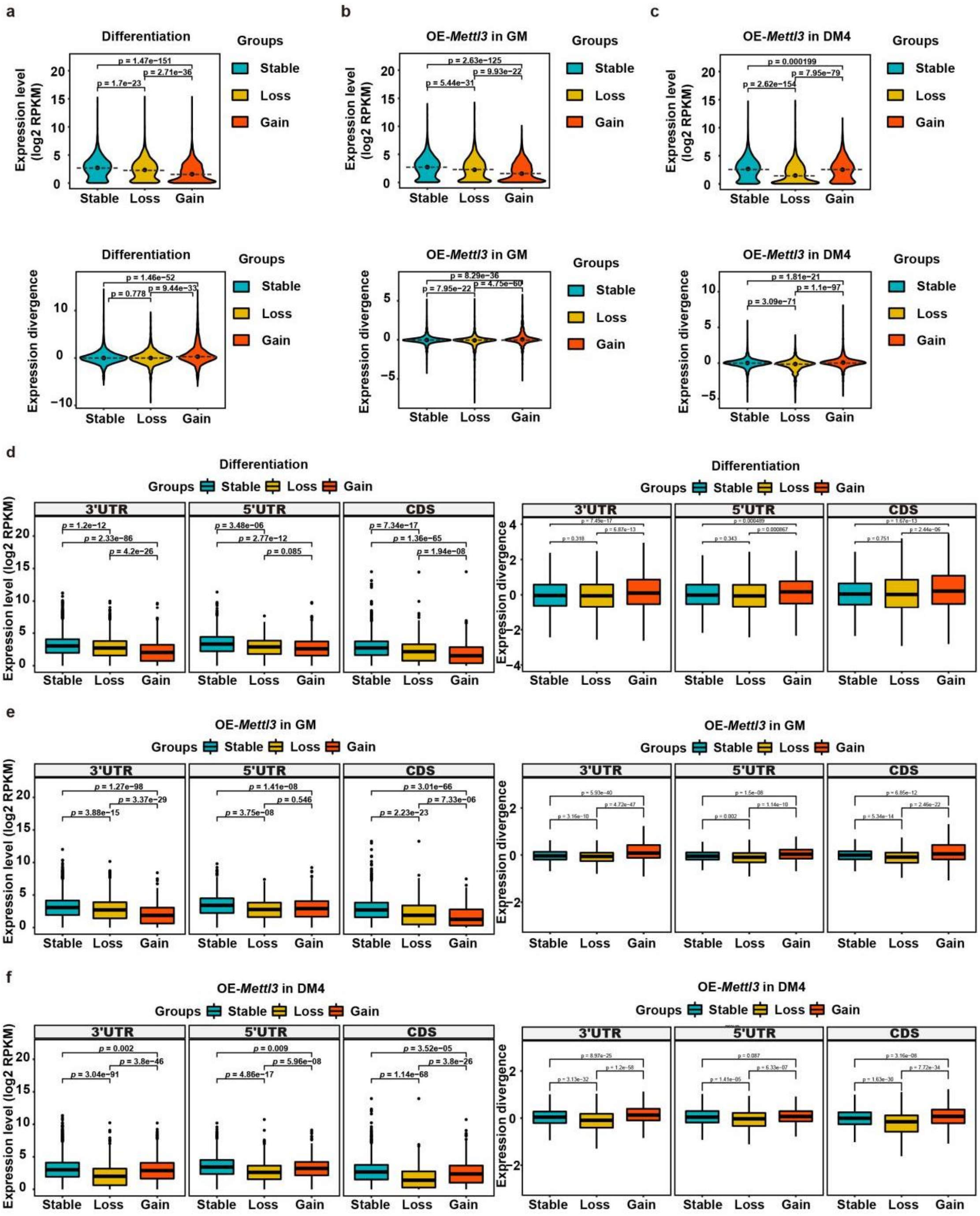

**Supplementary Fig 9. Link between RNA methylation dynamics and gene expression alterations.** **a** Violin plots showing the distribution of expression level and expression divergence of genes encoding mRNAs with common $m^6$As and specific $m^6$As in GFP-overexpressing cells during differentiation. **b** Violin plots showing the distribution of expression level and expression divergence of genes encoding mRNAs with common $m^6$As and specific $m^6$As in *Mettl3*-overexpressing cells and GFP-overexpressing cells prior to differentiation induction. **c** Violin plots showing the distribution of expression level and expression divergence of genes encoding mRNAs with common $m^6$As and specific $m^6$As in *Mettl3*-overexpressing cells and GFP-overexpressing cells on the fourth day post-differentiation. **d** Boxplots showing the expression level and expression divergence of genes encoding mRNAs with with common $m^6$As and specific $m^6$As at the 5'UTR, CDS, and 3'UTR of their transcripts in GFP-overexpressing cells during differentiation. **e** Boxplots showing the expression level and expression divergence of genes encoding mRNAs with with common $m^6$As and specific $m^6$As at the 5'UTR, CDS, and 3'UTR of their transcripts in *Mettl3*-overexpressing cells and GFP-overexpressing cells prior to differentiation induction. **f** Boxplots showing the expression level and expression divergence of genes encoding mRNAs with with common $m^6$As and specific $m^6$As at the 5'UTR, CDS, and 3'UTR of their transcripts in *Mettl3*-overexpressing cells and GFP-overexpressing cells on the fourth day post-differentiation. The rank sum test was used for statistical analysis in A, B and C. P values are indicated; RPKM, reads per kilobase of transcript per million mapped reads.

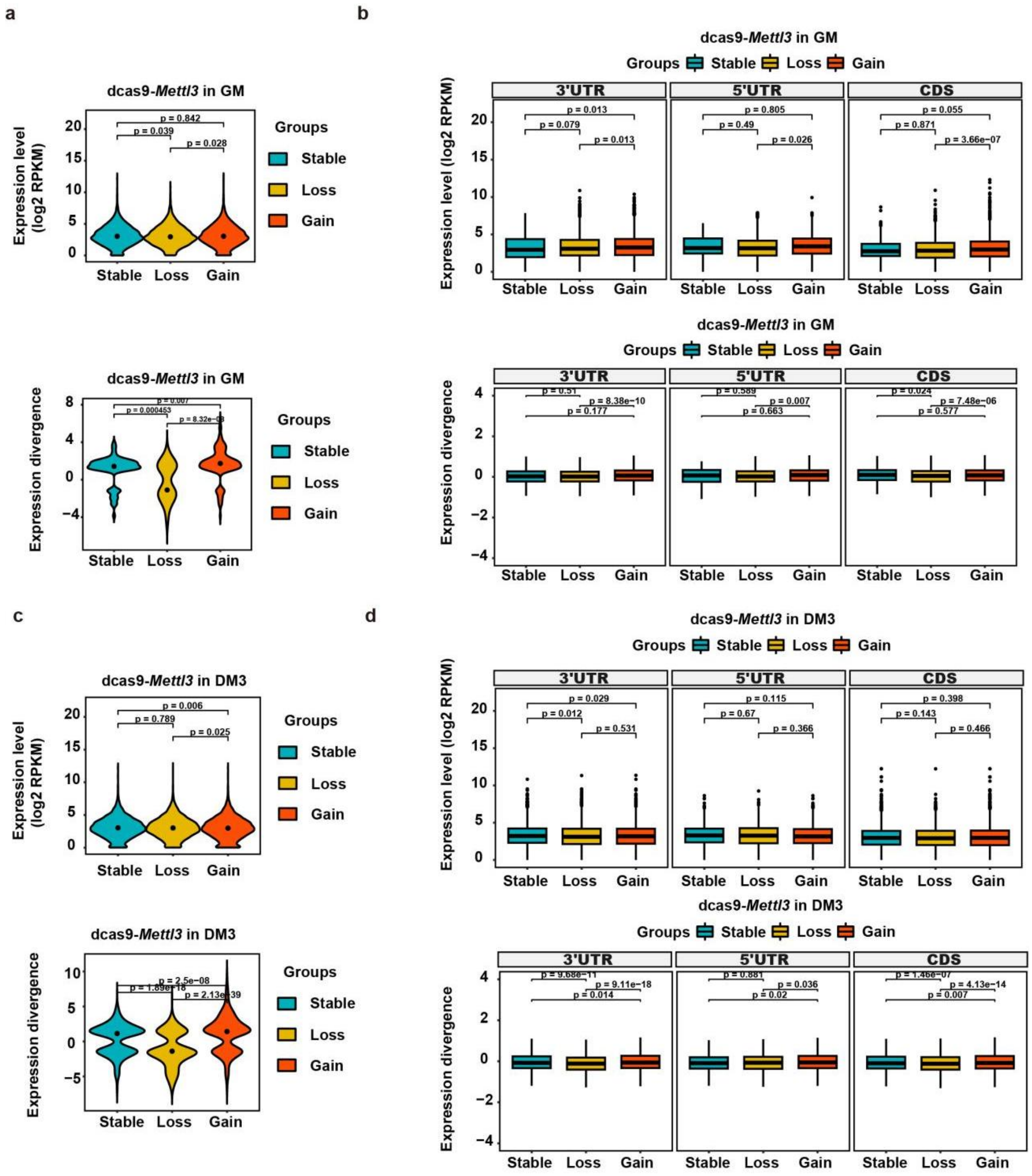


**Supplementary Fig 10. Link between RNA methylation dynamics and gene expression alterations. a** Violin plots showing the distribution of expression level and expression divergence of genes encoding mRNAs with common m$^6$As and specific m$^6$As in *Mettl3* knockdown cells compared to the control group prior to differentiation induction. **b** Boxplots showing the expression level and expression divergence of genes encoding mRNAs with with common m$^6$As and specific m$^6$As at the 5'UTR, CDS, and 3'UTR of their transcripts in *Mettl3* knockdown cells compared to the control group prior to differentiation induction. **c** Violin plots showing the distribution of expression level and expression divergence of genes encoding mRNAs

with common m⁶As and specific m⁶As in *Mettl3* knockdown cells compared to the control group on the third day post-differentiation. **d** Boxplots showing the expression level and expression divergence of genes encoding mRNAs with with common m⁶As and specific m⁶As at the 5'UTR, CDS, and 3'UTR of their transcripts in *Mettl3* knockdown cells compared to the control group on the third day post-differentiation. P values are indicated; RPKM, reads per kilobase of transcript per million mapped reads.

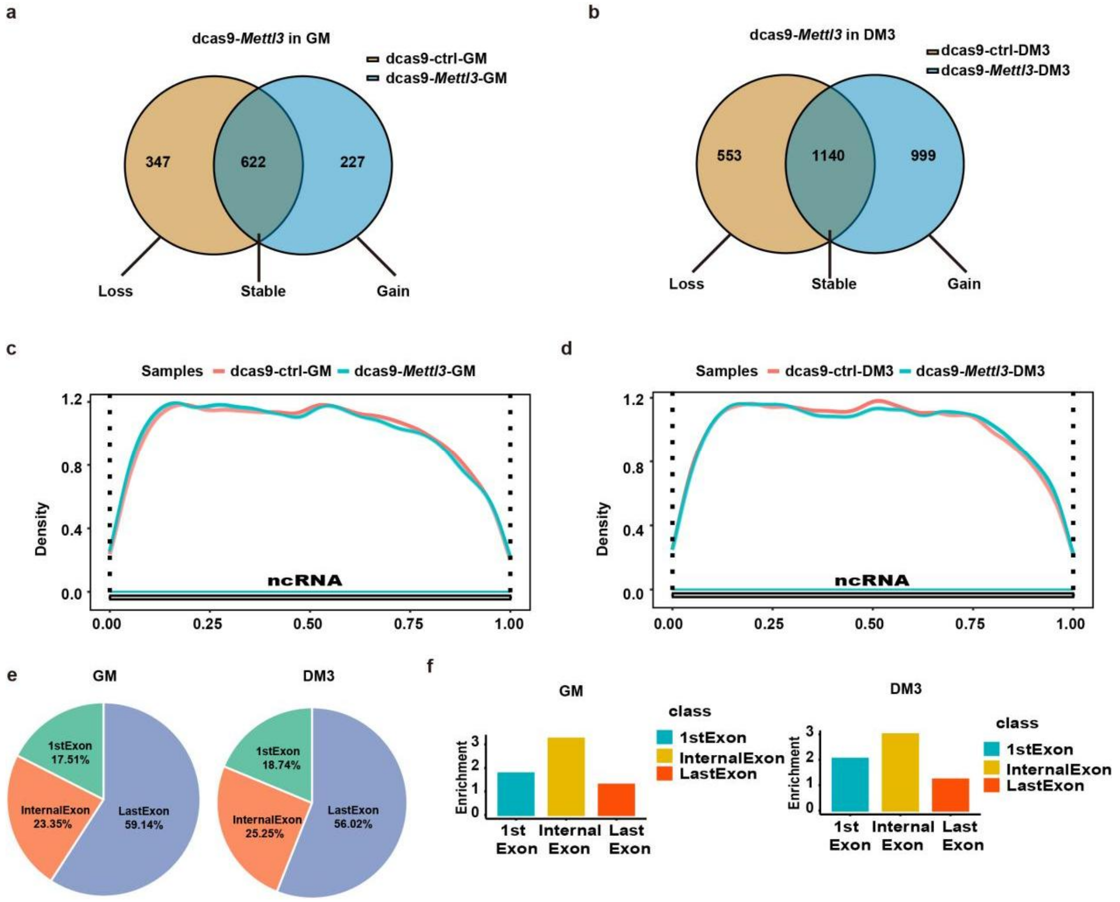


**Supplementary Fig 11. Features of METTL3 regulated lncRNA m⁶A alterations in *Mettl3* knockdown cells during differentiation.** **a** Venn diagram of lncRNA peaks enriched in *Mettl3* knockdown cells compared to the control group prior to differentiation induction. **b** Venn diagram of lncRNA peaks enriched in *Mettl3* knockdown cells compared to the control group on the third day post-differentiation. **c** Metagene profiles of enrichment of all m⁶A peaks across lncRNAs transcriptome in *Mettl3* knockdown cells compared to the control group prior to differentiation induction. **d** Metagene profiles of enrichment of all m⁶A peaks across lncRNAs

transcriptome in *Mettl3* knockdown cells compared to the control group on the third day post-differentiation. **e** Pie charts represent the proportion of $m^6A$ peaks in the three regions of lncRNAs before and after induced differentiation. **f** Histogram represents the relative enrichment of $m^6A$ peaks in the three regions of lncRNAs before and after induced differentiation.

a

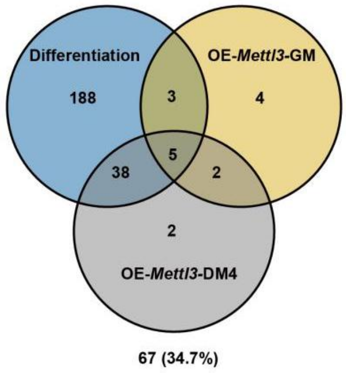


b

| M3_DM4VSGFP_DM4_M6A_new_fdr005 | | | GFP_DM4VSGFP_GM_M6A_new_fdr005 | | | |
|---|---|---|---|---|---|---|
| Gene Name | log2FC | FDR | Gene Name | log2FC | FDR | Differential change |
| 3300005D01Rik | 1.106868352 | 1.40E-13 | 3300005D01Rik | -0.738605253 | 5.68E-07 | 1.845473605 |
| Bc1 | 0.801513542 | 0.013009229 | Bc1 | -1.386133085 | 3.57E-07 | 2.187646627 |
| Snhg18 | 0.617025195 | 0.009410027 | Snhg18 | 0.860282025 | 0.000149459 | -0.243256829 |
| 4933404O12Rik | 0.59367684 | 0.037420981 | 4933413J09Rik | 2.441146162 | 2.27E-06 | -1.847469323 |
| Xist | -0.423720969 | 0.005073087 | Xist | 1.181915678 | 6.27E-20 | -1.605636647 |
| Gm37494 | -0.437230229 | 0.035887574 | Gm37494 | 0.96644978 | 4.02E-08 | -1.403680009 |
| Ftx | -0.551879276 | 0.004102711 | Ftx | 0.487068529 | 0.005252349 | -1.038947805 |
| Gm14635 | -0.67345712 | 0.005373935 | Gm14635 | 9.478458245 | 2.95E-10 | -10.15191537 |
| C030037D09Rik | -0.784642033 | 0.002264641 | C030037D09Rik | 1.622278316 | 7.32E-12 | -2.406920349 |
| Gm20342 | -0.789237707 | 0.048883634 | Gm20342 | 1.14568783 | 0.000951747 | -1.934925537 |
| Gm3830 | -0.818649357 | 0.004753683 | Gm3830 | 6.719594193 | 4.34E-18 | -7.53824355 |
| AI480526 | -0.832559187 | 0.010609397 | AI480526 | 2.411563239 | 3.82E-16 | -3.244122425 |
| Gm21781 | -0.879554565 | 0.000819172 | Gm21781 | 0.876863791 | 0.000249867 | -1.756418356 |
| Gm7967 | -0.937656496 | 0.002991306 | Gm7967 | 2.714950928 | 1.44E-17 | -3.652607424 |
| Gm45250 | -1.031816763 | 0.003005786 | Gm45250 | 1.351137814 | 1.86E-05 | -2.382954577 |
| Gm42047 | -1.033859325 | 0.031993482 | Gm42047 | -1.418534222 | 2.74E-05 | 0.384674897 |
| Gm20045 | -1.037898639 | 0.001812324 | Gm20045 | 1.501293145 | 7.51E-07 | -2.539191784 |
| Neat1 | -1.058803184 | 0.03553973 | Neat1 | 2.65023605 | 1.98E-10 | -3.709039234 |
| Gm47015 | -1.066291485 | 9.77E-08 | Gm47015 | 2.073636139 | 1.09E-28 | -3.139927624 |
| Gm32219 | -1.249519294 | 0.008846042 | Gm32219 | 1.791642663 | 4.14E-05 | -3.041161957 |
| 1700025L06Rik | -1.302364792 | 3.91E-06 | 1700025L06Rik | 5.062787459 | 3.23E-27 | -6.365152251 |
| Gm5532 | -1.36931549 | 0.032456276 | Gm5532 | 6.008782355 | 3.25E-07 | -7.378097845 |
| 2310001H17Rik | -1.392852585 | 0.001825661 | 2310001H17Rik | 2.698972064 | 1.61E-09 | -4.091824648 |
| 5730419F03Rik | -1.409102255 | 0.00030633 | 5730419F03Rik | 6.603086512 | 3.42E-16 | -8.012188767 |
| Gm28187 | -1.496047034 | 4.63E-05 | Gm28187 | 3.007712474 | 2.63E-14 | -4.503759508 |
| Gm6093 | -1.601230954 | 4.48E-09 | Gm6093 | 6.600023017 | 4.89E-51 | -8.201253971 |
| 4933413J09Rik | -1.683173019 | 0.002080818 | 4933404O12Rik | -2.110740583 | 6.83E-22 | 0.427567564 |
| 2310043L19Rik | -1.900835948 | 0.000176339 | 2310043L19Rik | 6.259516887 | 3.43E-08 | -8.160352835 |
| Gm37565 | -1.901467144 | 5.60E-07 | Gm37565 | 5.616399462 | 1.25E-16 | -7.517866606 |
| Gm19510 | -2.006995028 | 0.025746407 | Gm19510 | 2.929092438 | 0.000643135 | -4.936087466 |
| Gm34804 | -2.040775426 | 0.000768192 | Gm34804 | 8.240136861 | 1.78E-07 | -10.28091229 |
| G630018N14Rik | -2.287523567 | 0.028819454 | G630018N14Rik | 4.130652726 | 0.000418765 | -6.418176292 |
| Gm30551 | -2.308478497 | 0.00011727 | Gm30551 | 2.646400812 | 3.01E-06 | -4.954879309 |
| 2310065F04Rik | -2.379742775 | 1.46E-08 | 2310065F04Rik | 9.357033425 | 1.12E-09 | -11.7367762 |
| Gm14168 | -2.414645089 | 0.011940755 | Gm14168 | 5.844092941 | 0.000551376 | -8.25873803 |
| Gm38534 | -2.8515642 | 1.44E-09 | Gm38534 | 9.612877055 | 5.04E-10 | -12.46444125 |
| Gm33148 | -3.347526999 | 1.43E-11 | Gm33148 | 5.924586949 | 6.87E-13 | -9.272113948 |
| Gm36827 | -5.239369777 | 0.000115076 | Gm36827 | 6.359078492 | 0.000130382 | -11.59844827 |
| M3_GMVSGFP_GM_M6A_new_fdr005 | | | GFP_DM4VSGFP_GM_M6A_new_fdr005 | | | |
| Gene Name | log2FC | FDR | Gene Name | log2FC | FDR | Differential change |
| Gm2694 | 1.568888612 | 9.97E-08 | Gm2694 | -2.410388614 | 1.93E-11 | 3.979277226 |
| Gm28653 | -1.066101041 | 0.026715512 | Gm28653 | 6.761660182 | 4.47E-115 | -7.827761223 |
| Gm14221 | -1.407717604 | 0.000220001 | Gm14221 | 3.773255061 | 6.49E-68 | -5.180972664 |
| Gm35019 | -1.486293317 | 1.01E-10 | Gm35019 | 1.748195467 | 2.71E-18 | -3.234488784 |
| Gm38403 | -2.346661833 | 0.000820406 | Gm38403 | 4.526630426 | 4.09E-44 | -6.873292259 |

**Supplementary Fig 12. Features of METTL3 regulated lncRNA m$^6$A alterations during differentiation.**

**a** Venn diagram showing the number of overlapping DEGs between the three comparison groups during myoblasts differentiation and the number of differentially expressed transcripts in *Mettl3*-overexpressing cells and GFP-overexpressing cells before and after induced differentiation. **b** Analysis of the differential expression of m$^6$A-modified lncRNAs during myoblast differentiation and in *Mettl3*-overexpressing cells.

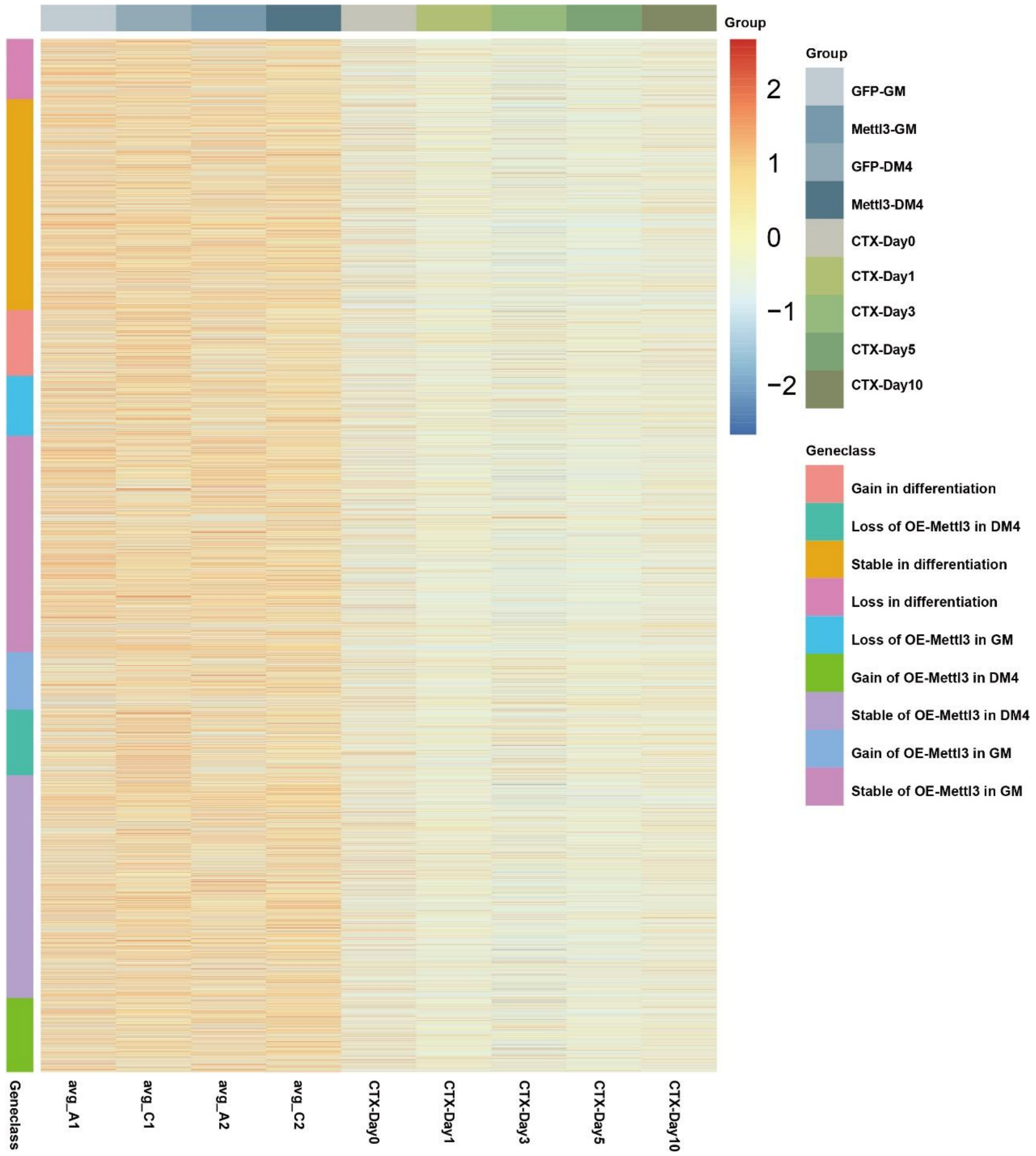


**Supplementary Fig 13. Bioinformatic cross-analysis of m$^6$A-mediated myogenesis-associated mRNAs.** Bioinformatic cross-analysis of m$^6$A-mediated myogenesis-associated mRNAs, from the mRNAs involved in skeletal muscle

regeneration, against all the m$^6$A-containing mRNAs previously identified by MeRIP-seq.

**Supplementary Fig 14. Unedited blot images of fig. 3a and fig. 3b.**

Fig. 3a

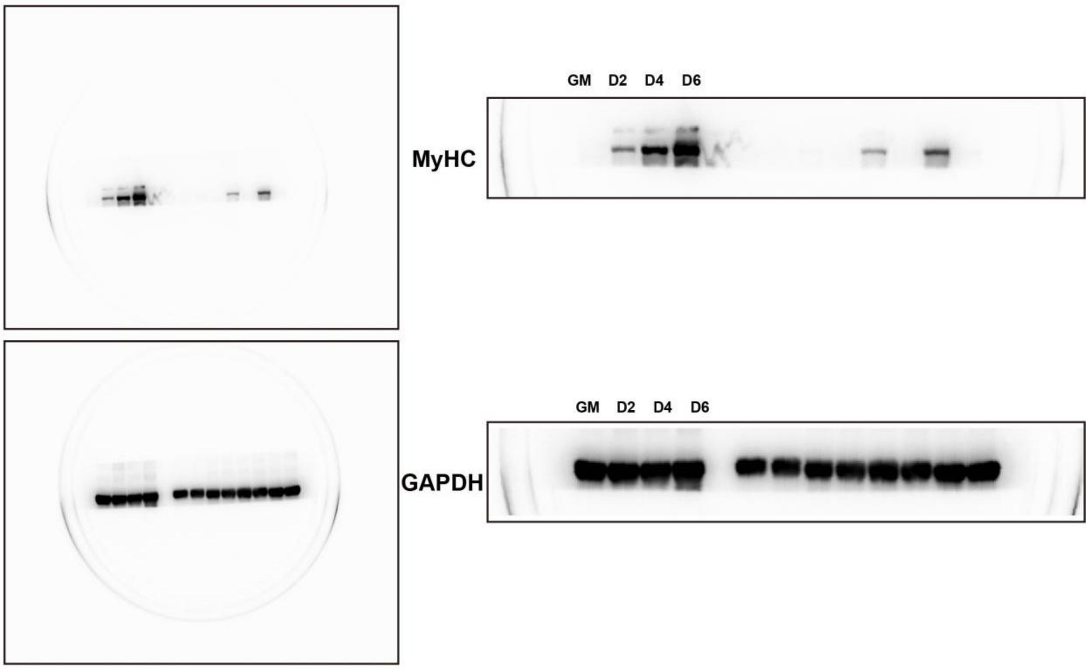


Fig. 3b

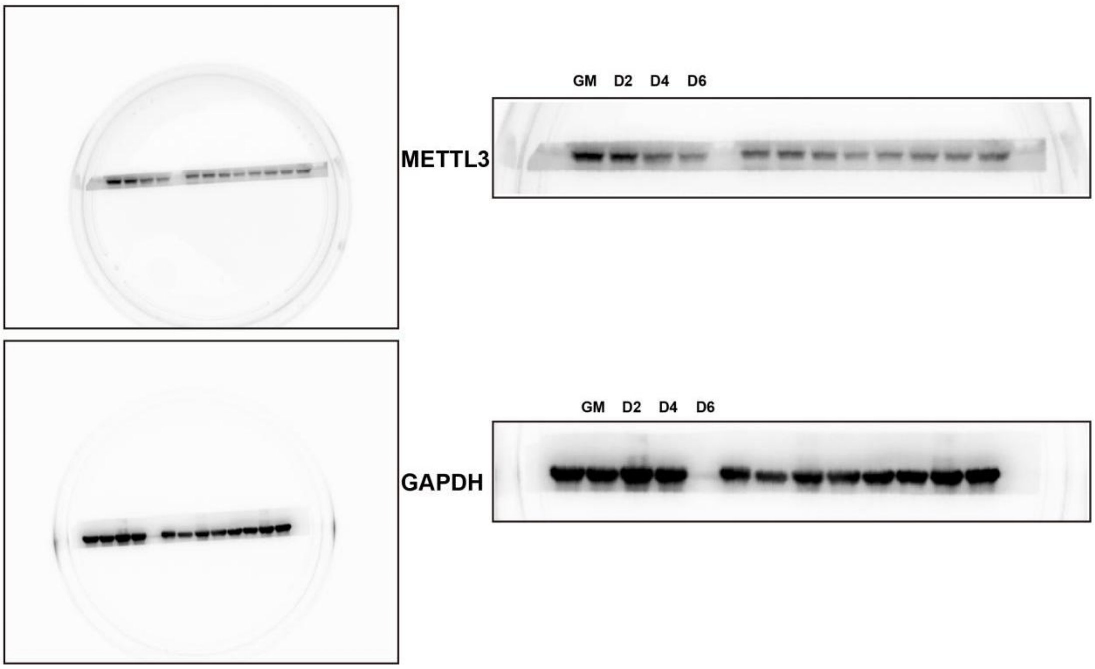


**Supplementary Fig 15. Unedited blot images of fig. 3c.**

Fig. 3c

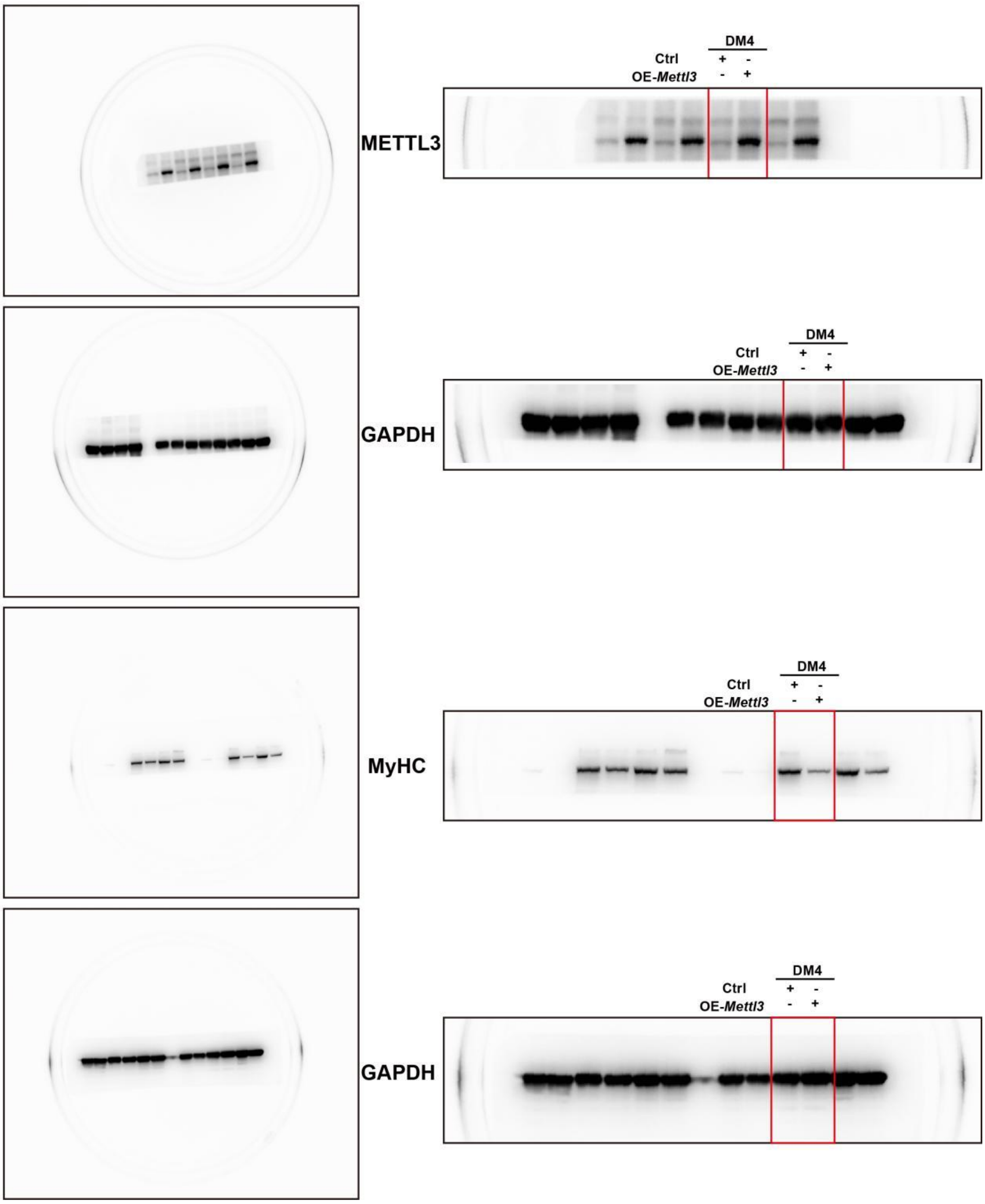


**Supplementary Fig 16. Unedited blot images of fig. 4a and fig. 4b.**

Figure4a

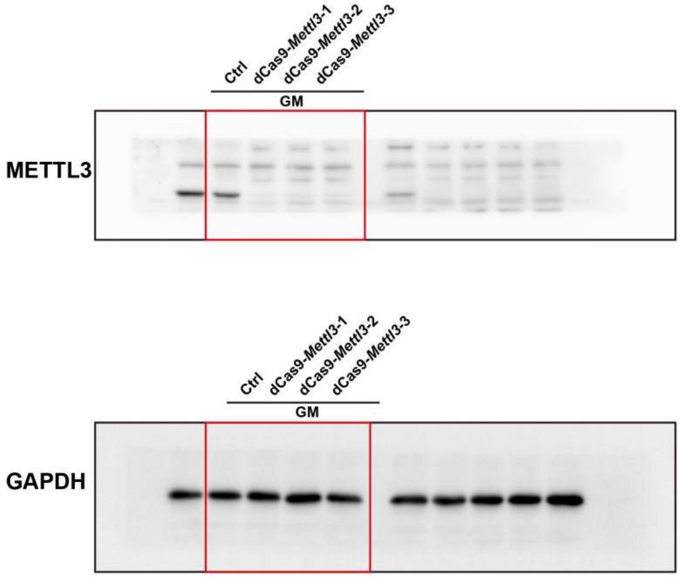


Figure4b

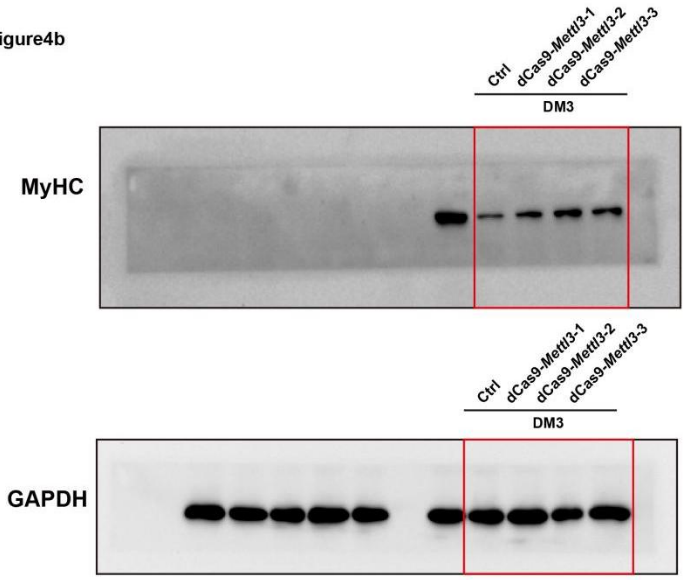

Supplement: Supplementary file 1 — Supplementary Information [file 42003_2025_8759_MOESM1_ESM.pdf]
