# Supplementary material for: Stage-specific requirement for METTL3-dependent m6A epitranscriptomic regulation during myogenesis
Source: Commun Biol. 2025 Aug 30;8:1317. doi: 10.1038/s42003-025-08759-5 (PMC12398539; doi:10.1038/s42003-025-08759-5)
Supplement: Supplementary file 15 — Description of Additional Supplementary Files [file 42003_2025_8759_MOESM15_ESM.pdf]

Description of Additional Supplementary Files

File name- Supplementary Data 1-

File description- Differential expression analysis of skeletal muscle regeneration

File name- Supplementary Data 2-

File description- GSEA analysis of the entire set of genes

File name- Supplementary Data 3-

File description- GSEA analysis of the entire set of genes-KEGG and GO analysis of overlapping genes

File name- Supplementary Data 4-

File description- Proteomic analysis

File name- Supplementary Data 5-

File description- Peak_fdr005

File name- Supplementary Data 6-

File description- Unique_peaks

File name- Supplementary Data 7-

File description- VENN analysis of m6 A-tagged mRNAs

File name- Supplementary Data 8-

File description- Differential expression analysis of m6 A-tagged mRNAs

File name- Supplementary Data 9-

File description- Fuctional analysis of m6 A-tagged mRNAs

File name- Supplementary Data 10-

File description- Differential expression analysis of m6 A-tagged LncRNAs

File name- Supplementary Data 11-

File description- VENN analysis of m6 A-tagged LncRNAs

File name- Supplementary Data 12-

File description- Bioinformatic cross-analysis of m6A-mediated myogenesis-associated mRNAs
